# Evaluating the convergence between eddy-covariance and biometric methods for assessing carbon budgets of forests

**DOI:** 10.1038/ncomms13717

**Published:** 2016-12-14

**Authors:** M. Campioli, Y. Malhi, S. Vicca, S. Luyssaert, D. Papale, J. Peñuelas, M. Reichstein, M. Migliavacca, M. A. Arain, I. A. Janssens

**Affiliations:** 1 Centre of Excellence PLECO (Plant and Vegetation Ecology), Department of Biology, University of Antwerp, 2610 Wilrijk, Belgium; 2 Environmental Change Institute, School of Geography and the Environment, University of Oxford, Oxford OX1 3QY, UK; 3 LSCE CEA-CNRS-UVSQ, Orme des Merisiers, F-91191 Gif-sur-Yvette, France; 4 DIBAF, University of Tuscia, 01100 Viterbo, Italy; 5 Euro-Mediterranean Center on Climate Change, CMCC, 73100 Lecce, Italy; 6 CSIC, Global Ecology Unit, CREAF-CEAB-CSIC-UAB, Cerdanyola del Vallès, 08193 Barcelona, Catalonia, Spain; 7 CREAF, Cerdanyola del Vallès, 08193 Barcelona, Catalonia, Spain; 8 Max Planck Institute for Biogeochemistry, 07745 Jena, Germany; 9 School of Geography & Earth Sciences, McMaster University, Hamilton, Ontario, Canada L8S 4K1

## Abstract

The eddy-covariance (EC) micro-meteorological technique and the ecology-based biometric methods (BM) are the primary methodologies to quantify CO_2_ exchange between terrestrial ecosystems and the atmosphere (net ecosystem production, NEP) and its two components, ecosystem respiration and gross primary production. Here we show that EC and BM provide different estimates of NEP, but comparable ecosystem respiration and gross primary production for forest ecosystems globally. Discrepancies between methods are not related to environmental or stand variables, but are consistently more pronounced for boreal forests where carbon fluxes are smaller. BM estimates are prone to underestimation of net primary production and overestimation of leaf respiration. EC biases are not apparent across sites, suggesting the effectiveness of standard post-processing procedures. Our results increase confidence in EC, show in which conditions EC and BM estimates can be integrated, and which methodological aspects can improve the convergence between EC and BM.

## Abstract (web summary)

Site-level quantification of Net Ecosystem Production (NEP) and associated components rely on eddy covariance and biometric methods. Here these techniques are compared for global forest carbon fluxes, revealing differences in NEP, but similar estimates of ecosystem respiration and gross primary production.

The exchange of carbon dioxide (CO_2_) between terrestrial ecosystems and the atmosphere is one of the major interactions between the biosphere and the atmosphere (Fig. 1), a key descriptor of ecosystem functioning and a major influence on atmospheric CO_**2**_ concentration. Two empirical approaches are generally used to quantify ecosystem CO_2_ exchange at the ecosystem level: the eddy-covariance technique (EC) and biometric methods (BM).

The EC technique features sound underlying micro-meteorological principles, continuous monitoring, little perturbation or damaging of the system sampled and a sampling area (footprint) well suited for the scale of ecosystem-level estimates (Table 1). The long time series with high temporal resolution generated by EC can give detailed insights into the interactions between CO_2_ fluxes and synoptic and seasonal variability. Therefore, EC is very attractive for long-term monitoring of the net ecosystem-atmosphere CO_**2**_ exchange^1^ (or net ecosystem production^2^, NEP) and for the elucidation of its temporal changes and environmental controls. These properties have made EC the dominant methodology for estimating net and bulk fluxes of CO_2_ exchange^1^,^3^,^4^ and the standard method in a number of long-term and large-scale research infrastructures (for example, ICOS, NEON, AmeriFlux, TERN). However, as with every experimental method, EC has some drawbacks (Table 1), three of which are of particular importance. First, advective and low-frequency flows of CO_2_ are difficult to capture and can potentially lead to underestimation of fluxes during periods with low air turbulence, typically ecosystem respiration at night^5^. This drawback is particularly important in the presence of variable topography, favouring air drainage and breezes^6^, or thick canopy, hindering mixing of the air within and above it^7^,^8^. Second, EC has a persistent inability to close the surface energy budget, leading to fears that if energy fluxes are being underestimated, then CO_2_ fluxes may also be underestimated^1^. Third, EC measures NEP directly, but its two main components, ecosystem photosynthetic CO_2_ uptake, or gross primary production (GPP), and ecosystem carbon (C) release, or ecosystem respiration (Reco) (Fig. 1), can only be estimated indirectly by post-processing the data of CO_2_ exchange^9^,^10^. In other words, EC relies on a single measurement to estimate net and bulk CO_2_ fluxes.

The BM approach uses a well-established but un-standardized set of techniques, such as plant growth assessment, chamber-based flux measurements and repeated stock inventories that allow a direct estimation of the component processes of the ecosystem C cycle (for example, net primary production (NPP), heterotrophic respiration (Rh) and autotrophic respiration (Ra); Fig. 1; Table 1) and changes in soil and biomass stock, from which NEP, Reco and GPP can be calculated. Advantages of this approach include insights into the internal C dynamics of an ecosystem, (for example, partitioning between Ra and Rh, allocation of photosynthates between Ra and NPP and allocation of NPP between leaves, wood and fine roots), and applicability to almost any site (for example, small plots, sites with strong spatial heterogeneity, high canopy thickness or steep topography) and meteorological conditions (for example, periods with low air turbulence) without the requirements imposed by the EC technique. Typically, BM approaches are also very useful for evaluating the impact of environmental manipulative experiments on the C cycle^11^, whereas EC cannot be applied to experimental plots of limited size^5^. On the other hand, BM approaches also have drawbacks (Table 1). In particular, biometric measurements are typically performed on few replicated individuals and plant organs (for example, few leaves and branches) or small ecosystem plots that need to be up-scaled, assuming homogeneity within and among plants and in all relevant environmental variables (for example, soil moisture, nutrients, microclimate, soil type). Moreover, there is always the possibility that some potentially important components of the C budget have not been accounted for (for example, transfer of photosynthates to mycorrhizae production, ground flora productivity or loss to herbivory) and that some of the biometric techniques can disturb the portion of the ecosystem being sampled (for example, root measurements disturb the soil, stem respiration chambers can affect microclimate and pressure of the air space sampled). Finally, most biometric measurements cannot be easily monitored continuously, making the linkage between changes in fluxes to specific weather events more challenging.

As the advantages of BM (for example, applicability to most sites and environmental conditions) largely match the potential disadvantages of the EC technique (and vice versa) and the two techniques are fully independent, the comparison between EC and BM has been developed as the most suited way to corroborate both approaches^12^. NEP estimates obtained with EC and BM have been compared in a number of studies, but no clear picture has yet emerged. Agreement of multi-year NEP estimates between methods varied widely among sites, from very good^13^ to very poor^14^. A primary cause of our limited understanding of EC–BM convergence lies in the fact that existing empirical studies are based only on one or few sites (for example, five sites^12^) and very few studies have attempted quantitative multi-site syntheses^15^. In practice, this has made it difficult to pin-point the reasons behind the observed cases with low convergence because statistical analyses have not been undertaken yet. Comparability of BM and EC estimates for Reco and GPP has been studied even less than for NEP. We are not aware of any multi-site synthesis efforts and existing comparisons are limited to 3–4 sites within the same region^16^,^17^. Lack of knowledge about EC and BM comparison for Reco and GPP complicates the analysis of NEP estimates obtained by the two approaches (for example, the two approaches could give similar NEP while diverging in their estimation of the two components). As a result of the multiple limitations on the corroboration of the empirical estimates of NEP, Reco and GPP, our understanding of the uncertainty of ecosystem C fluxes has stagnated for over a decade: the suspected biases of EC have not been clarified^18^ and the uncertainties in BM estimates remain underexplored.

Here we investigate the agreement between EC- and BM-based estimates of ecosystem CO_2_ fluxes by addressing three research questions: how do EC and BM-based fluxes compare across and within the boreal, temperate and tropical forest zones? Is any discrepancy between EC and BM flux estimates related to stand, environmental and methodological variables? Can the EC–BM comparison provide insights into long-standing suspected biases of the EC technique? The answers are provided by analyzing a novel EC and BM data set comprising annual estimates of NEP, Reco and GPP for 40 sites across five continents, spanning boreal, temperate and tropical forests. The convergence between EC and BM fluxes is analysed through the absolute and relative flux difference, globally and for the three climatic zones separately, and by testing the correlation of the flux differences with a large set of stand, environmental and methodological variables (for example, indices of topographical complexity, leaf are index, mean annual temperature, approaches to scale up the tree respiration). It is found that EC and BM provide globally comparable estimates of Reco and GPP but different NEP, which is smaller and more susceptible to biases. Moreover, biases of opposite direction are likely to cancel out when estimating Reco and GPP. Low convergence is associated with BM approaches underestimating NPP and neglecting light inhibition of leaf dark respiration, with discrepancies more pronounced for the boreal zone. Major EC biases are not apparent across sites, increasing our confidence in this technique.

## Results

### Data set of process components of ecosystem carbon cycle

Our uniform and quality-checked data set of BM and EC-based forest C fluxes comprised 31 sites with BM-based NEP (NEP_BM_) and EC-based NEP (NEP_EC_), 25 sites with BM-based Reco (Reco_BM_) and EC-based Reco (Reco_EC_) and 18 sites with BM estimates of GPP (GPP_BM_) and EC estimates of GPP (GPP_EC_) (Supplementary Tables 1–4; Supplementary Fig. 1; Supplementary Data 1). About 60–70% of the sites were in the temperate zone, 20–25% in the boreal zone and 10–15% in the tropical zone (Table 2). NEP ranged from −110 to 830 gC m^−2^ y^−1^, Reco from 460 to 3,300 gC m^−2^ y^−1^, and GPP from 600 to 3,600 gC m^−2^ y^−1^ (EC data). Furthermore, BM data allowed insights into the different components of the C cycle of the studied forests. First, NPP ranged from 170 to 1,500 gC m^−2^ y^−1^, Ra from 490 to 1,900 gC m^−2^ y^−1^ whereas soil heterotrophic respiration (Rh-soil) from 170 to 1,400 gC m^−2^ y^−1^. Second, leaves, aboveground wood and roots accounted on average for 30±2% (mean and s.e.m), 34±2% and 30±3% of NPP, respectively, and 39±4%, 22±3% and 38±4% of Ra, respectively. Third, fine roots accounted for 70±7% of root NPP. Fourth, Reco was composed of 55±3% soil respiration (Rsoil), 28±2% leaf respiration (Rleaf), 15±2% aboveground wood respiration (Rwood) and 5±1% heterotrophic respiration of coarse woody debris (Rh-cwd), that is, 68±3% by Ra and 32±3% by Rh.

### Net ecosystem production

**Figure 2:**
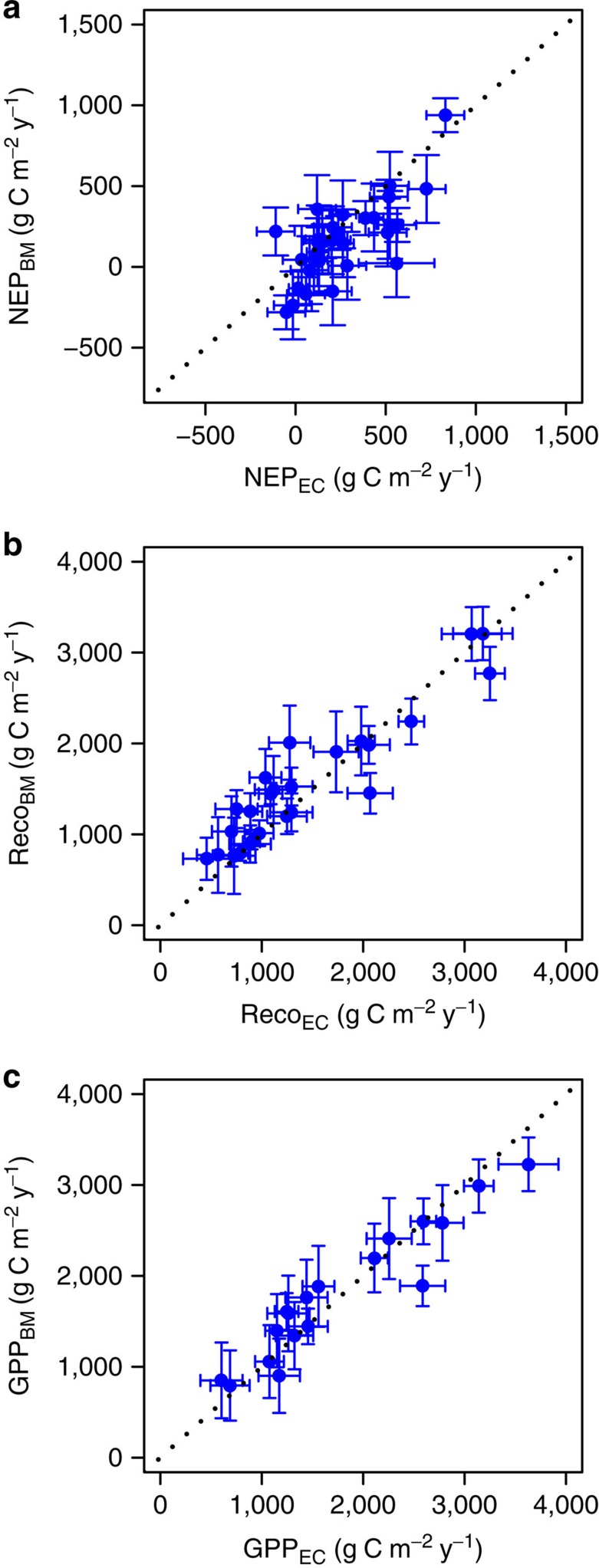
Comparison of carbon fluxes obtained from eddy-covariance or biometric methods for worldwide forests. (**a**) Net ecosystem production (NEP, *n*=31), (**b**) ecosystem respiration (Reco, *n*=25) and (**c**) gross primary production (GPP, *n*=18) from eddy-covariance (EC; *x* axis) and biometric (BM; *y* axis) methods. Bars indicate confidence intervals which are derived from uncertainty ranges related to biome and latitude, constrained by a reduction factor depending on the methodology and by the number of replicate years of measurement (see Methods). The dotted line is the 1:1 line.

NEP_BM_ agreed with NEP_EC_ along the range of flux measurements as indicated by the slope of the regression NEP_BM_ versus NEP_EC_ (1.06; CI_95%_=0.74–1.53, *R*^2^=0.54) (Fig. 2a). However, NEP_EC_ was significantly larger than NEP_BM_ (*P*<0.01; mean difference 98±32 gC m^−2^ y^−1^; Table 2). This trend was caused by the extratropical sites, particularly in the boreal zone, where the mean difference was as large as 167±44 gC m^−2^ y^−1^ (Table 2).

The difference between NEP_EC_ and NEP_BM_ was not correlated to the elevation variability, topographical slope and leaf area index (LAI), or to any of the other environmental and stand variables considered (Table 3). Similarly, the difference between NEP_EC_ and NEP_BM_ was not correlated to the type of BM approach applied (that is, different methods to measure fine root NPP, leaf NPP and Rh-soil, different quality of the allometric relationships used to estimate wood NPP and accounting or not for Rh-cwd; Table 3; Supplementary Fig. 2). Finally, the difference between NEP_EC_ and NEP_BM-ΔS_ (BM-based NEP derived from the difference in ecosystem C stocks between two points in time, thus avoiding the use of NPP and Rh data, see Methods and Supplementary Table 5) was also not statistically significant (Table 2).

### Ecosystem respiration

The regression between Reco_BM_ and Reco_EC_ had a slope of 0.86 (CI_95%_=0.73–1.02; *R*^2^=0.87) (Fig. 2b). Reco_BM_ was on average 13±4% larger than Reco_EC_ (marginally significant at *P*=0.061). Although the differences between EC- and BM-based estimates of Reco did not present any correlation with the indices of topographical complexity, LAI or any other environmental or stand variables (Table 3), Reco_BM_ and Reco_EC_ were significantly different for the boreal forests (difference 18±7%, *P*=0.031) but were not significantly different for the temperate (16±6%, *P*=0.079) and tropical forests (5±5%, *P*=0.39) (Table 2).

**Figure 3:**
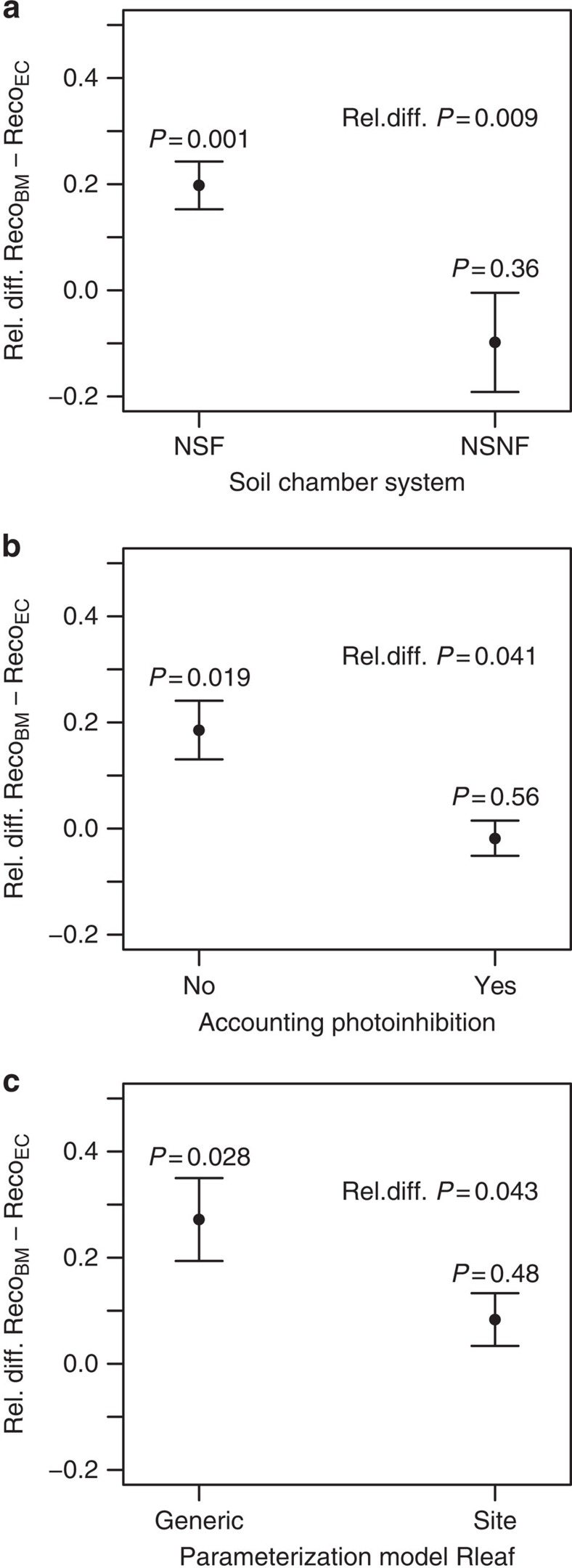
Impact of variants of biometric methods on the difference between ecosystem respiration from biometric methods and eddy-covariance. The relative difference between ecosystem respiration from biometric methods (Reco_BM_) and eddy-covariance (Reco_EC_) [(Reco_BM_ − Reco_EC_)/((Reco_EC_ + Reco_BM_)/2)] when (**a**) using different chamber systems to measure soil respiration (NSF: non-steady-state through-flow chamber; NSNF: non-steady-state non-through-flow chambers), (**b**) whether light inhibition is accounted for when estimating leaf respiration (Rleaf), and (**c**) employing generic or site-specific parameterization for the empirical models used to scale up the point measurements of Rleaf to the annual scale. Points indicate means and bars the standard error of the mean. The *P* value above each point indicates the significance level of the difference between Reco_BM_ and Reco_EC_ for each case, whereas the significance level *P* of each factor (that is, chamber system, accounting light inhibition, parameterization type) is indicated as rel. diff. *P* (relative difference between Reco_BM_ and Reco_EC_) and is reported in the top right of each panel.

The degree of the convergence between Reco_BM_ and Reco_EC_ differed according to the type of chamber technique used to measure Rsoil. For sites where closed static chambers or non-steady-state non-through-flow chambers (NSNF) were used, Reco_BM_ and Reco_EC_ did not differ significantly (*P*=0.36; Fig. 3a). On the other hand, the use of closed dynamic chambers or non-steady-state through-flow chamber (NSF) resulted in Reco_BM_ significantly larger than Reco_EC_ (20%, *P*<0.001; Fig. 3a). Within the NSF group, the low level of convergence between Reco_BM_ and Reco_EC_ did not differ (*P*=0.22) when standard systems and systems prescribing scrubbing of CO_2_ before the flux measurements (for example, Li-Cor LI-6400-09 system; LI-COR Biosciences, Lincoln, USA) were compared (Table 3). Also the BM approach used for measuring Rleaf had an impact on the convergence between Reco_BM_ and Reco_EC_. Neglecting light inhibition of leaf dark respiration increased the difference between methods by about 20% (*P*=0.041; Fig. 3b; Table 3). Likewise, models used to scale up point measurements of Rleaf to annual values also increased the difference between methods by about 20% when they had a generic formulation that is, without a site-specific parameterization (*P*=0.043; Fig. 3c; Table 3). The different chamber systems for measuring Rsoil affected the convergence between Reco_BM_ and Reco_EC_ also when their effect was disentangled from the effect of light inhibition and parametrization type of leaf respiration (Supplementary Fig. 3). On the other hand, the effect of neglecting light inhibition was stronger than the parameterization type as it was the only one significant when the two effects were disentangled. In fact, a two-way analysis of variance (ANOVA) accounting for Rleaf light inhibition (yes/no) and parametrization type (site-specific/generic), performed only for sites with NSF measurements, showed that only light inhibition was significant (*P*=0.035; parametrization type: *P*=0.23; interaction term: *P*=0.70).

No impact was observed on the convergence between Reco_BM_ and Reco_EC_ from other technical variants in measuring Reco_BM_. The latter included: considering (or neglecting) Rh-cwd and the growth respiration of Rleaf and Rwood; the differentiation of Rwood in stem and branch respiration; the types of model drivers (temperature or others) used for the annual estimation of Rleaf, Rwood and Rsoil; the variability of the temperature sensitivity of models of Rleaf, and the type of upscaling variable used for Rwood (wood volume or wood area) (Table 3). Similarly, the partitioning method used to derive Reco_EC_ from NEP_EC_ did not have a major impact on the difference between Reco_EC_ and Reco_BM_. The use of the daytime derived Reco instead of night time derived Reco decreased the divergence between Reco_EC_ and Reco_BM_ (from 13±7% to 9±4%). However, this improvement was not statistically significant (*P*=0.29) because daytime derived Reco_EC_ did not differ significantly from night time derived Reco_EC_ (4±4%, daytime Reco_EC_>night time Reco_EC_; *P*=0.64).

### Gross primary production

The regression of GPP_BM_ versus GPP_EC_ had a slope of 0.84 (CI_95%_=0.70–0.99; *R*^2^=0.90). The agreement between methods was confirmed by the small and non-significant relative difference between GPP_BM_ and GPP_EC_ (5±4%). The mean difference between GPP_BM_ and GPP_EC_ was small (up to 8%) and non-significant also when the analysis was performed for the three climatic zones separately (Table 2). As for NEP and Reco, the differences between EC- and BM-based estimates of GPP were not related to any environmental or stand variables (Table 3). The relationship between the GPP_BM_ and GPP_EC_ difference and the topographical slope was marginally significant, but this was due to one outlier (Supplementary Fig. 4; the relationship was not significant at *P*=0.19 when the outlier was removed) and was therefore considered irrelevant. The impact of different BM approaches to measure NPP, Rleaf and Rwood did not have any significant impact on the difference between GPP_EC_ and GPP_BM_, except for a minor effect of the variables used to model Rwood (*P*=0.086) (Table 3). As for Reco, the use of daytime flux data, instead of night time flux data, for the calculation of GPP_EC_ (GPP_EC_=Reco_EC_+NEP_EC_) improved the difference between GPP_EC_ and GPP_BM_ (from 5±5% to 2±2%). However, this change was non-significant (*P*=0.81) mainly because daytime-data derived GPP_EC_ did not differ than night time-data derived GPP_EC_ (2±2%, daytime GPP_EC_ > night time GPP_EC_, *P*=0.91).

## Discussion

Here we will discuss four major points: the insight that our analysis provides on EC and on BM, the low convergence between NEP_EC_ and NEP_BM_, the EC–BM convergence for Reco and GPP and the different degree of the methodological convergence observed for NEP, Reco and GPP.

As no recognized reference method exists for CO_2_ exchange in ecosystems, in principle, it is not possible to evaluate the correctness of EC in measuring NEP, and in assessing Reco and GPP. However, our study provided several indirect indications about the general reliability of EC-based estimates of C fluxes. For example, the lack of correlation of the difference between EC- and BM-based estimates of C fluxes versus site topographical complexity and LAI suggests that the standard post-processing procedures used to account for advective and low-frequency flows of CO_2_ are effective. In fact, without the appropriate amendment, increasing site topographical complexity and LAI should increase the chances of advective and low-frequency flows of CO_2,_ causing biases in EC estimates^5^,^6^. These issues, which can be relevant for particular sites^8^,^18^, are therefore less important at multiple-site level. Another indication about the reliability of EC-based C fluxes is that, estimates of Reco_EC_ (and GPP_EC_) based on daytime flux data do not differ significantly from estimates of Reco_EC_ (and GPP_EC_) based on night time flux data (as was also demonstrated elsewhere^9^), and their use here does not improve the convergence between EC and BM. This specifically supports that problems related to low-turbulence conditions during night are in general sufficiently amended by the flux data treatment^5^. Finally, the lack of major divergence between night time estimated Reco_EC_ and Reco_BM_ further reduce the probability that estimates of NEP_EC_ are affected by major night time overestimation, at least for temperate and tropical forests.

Contrary to EC, no standard procedures exist for BM and the BM studies included in our synthesis presented different approaches in determining the production and respiration components of the ecosystem C cycle. The methodological variation in BM was important and allowed us to detect which approach best converged with EC. Measurements of respiration were particularly affected by the methodological approach. For Rsoil (which represents the dominant component of Reco), it is known that the NSNF chamber system significantly underestimates Rsoil, whereas the NSF system presents smaller inaccuracies^19^. This was indirectly reflected by our results. As Reco_BM_ was overall larger than Reco_EC_, the agreement of these estimates when the NSNF system was used indicates a potential case of error compensation, with the underestimation of belowground respiration compensating an overestimation of aboveground respiration (see below). On the other hand, the latter term was not compensated when the NSF was used, with Reco_BM_ having larger values than Reco_EC_ at sites making use of NSF. Leaf respiration is also an important component of Reco (about 30%). Our data show that, the consideration or not in BM of light inhibition of daytime Rleaf (it was considered in only 28% of our sites) has a major influence on the convergence between Reco_EC_ and Reco_BM_. This is striking as light inhibition of daytime Rleaf is also neglected in standard (night time derived) estimates of Reco and might not be correctly accounted for even in the daytime derived Reco^20^. A possible explanation is that error compensation might reduce the impact of neglecting light inhibition of Rleaf more in EC- than BM-based estimates (for example, the overestimation caused by not accounting for the light inhibition of Rleaf might be compensated by missing fluxes), and should be examined further. On the other hand, our analysis did not show major issues related to scaling in BM-based estimates of respiration. For instance, Rwood, the Rwood:Reco ratio and the convergence between Reco_BM_ and Reco_EC_ were not related to the scaling variable used for the wood respiration rate (stand wood volume or wood surface area, *P*=0.3–0.8; Table 3; Supplementary Table 6). On the other hand, for the leaf respiration rate that is typically scaled up using estimates of LAI, Rleaf, the Rleaf:Reco ratio and the convergence between Reco_BM_ and Reco_EC_ did not vary between needleleaved and broadleaved species (*P*=0.2–0.3; Table 3; Supplementary Table 6). This is the case even though the leaf area estimates of needle-leaved species are more prone to biases than for broadleaved species^21^.

Methodological variants in measuring NPP did not show any significant impact on the convergence between EC and BM. For wood, flaws in allometric relationships can cause either overestimation or underestimation of NPP^22^ and thus errors are compensated when multiple sites are considered. For leaves, the use of the litter trap method did not seem to provide different production estimates for deciduous or evergreen stands, as they show practically the same leaf-to-aboveground production ratio (0.45±0.03 and 0.45±0.02, respectively; *P*=0.99). On the other hand, the use of litter traps or allometric relationships resulted in different leaf-to-aboveground production ratio in evergreen stands (0.32±0.06 and 0.45±0.02, respectively; *P*=0.022). This indicates that these two methodologies may provide different estimates of leaf NPP for evergreen species but also that this systematic difference is too small to affect the EC–BM convergence for NEP and GPP (Table 3). For fine roots, our results did not show the typical bias pattern associated with the different methods to measure NPP (for example, underestimations for ingrowth cores and overestimation for minirhizotron^23^), which may be due to the overall large uncertainty in fine root NPP estimates.

Our study generally finds good support for the reliability and consistency of EC and BM approaches to estimate forest C fluxes at the ecosystem level. However, the low convergence recorded between NEP_EC_ and NEP_BM_, with NEP_EC_ significantly larger than NEP_BM_ for extratropical forests, is an important exception. For temperate forests, the mean difference was 100 gC m^−2^ y^−1^, which is broadly comparable to the inter-annual variability for this biome^13^,^17^,^24^, characterized by NEP of about 300–400 gC m^−2^ y^−1^ (ref. 25). For the boreal zone, the mean difference was about 170 gC m^−2^ y^−1^, which is sufficiently large to confound the ecosystem sink-source status as NEP of boreal forests is typically between 40 and 180 gC m^−2^ y^−1^ (ref. 25). For instance, the mean NEP_BM_ of the boreal sites in our data set was −60±60 gC m^−2^ y^−1^ (C source) whereas the mean NEP_EC_ was 110±40 gC m^−2^ y^−1^ (C sink). The reason for this pattern is not clear, as we did not observe significant relationships between the low convergence between NEP_EC_ and NEP_BM_ and climatic or environmental variables (such as mean annual temperature, mean annual precipitation and site fertility, which are all lower in the boreal zone). Probably, the relatively small C fluxes in boreal forests make them more susceptible to methodological biases.

As we did not find any indications for major overestimations of NEP_EC_, it is relevant to explore if the dominant BM approach used to measure NEP is affected by any systematic underestimation that could explain the difference between NEP_EC_ and NEP_BM_. As the usual approach to estimate NEP_BM_ is by subtracting heterotrophic respiration (Rh-soil and Rh-cwd) from NPP, underestimation of NEP_BM_ could be caused by overestimation of Rh-soil and Rh-cwd or underestimation of NPP. Rh-cwd is a relatively small flux in most sites and we have not recorded lower agreement between NEP_BM_ and NEP_EC_ for sites missing this component of the C cycle. Ecosystem Rh-soil is particularly difficult to measure and, in our data set, four major measurements approaches were used: root exclusion to estimate Rh-soil (60% of sites), measuring Rroot and then subtracting it from Rsoil (20% of sites), calculating Rh-soil from separate incubation of all components of the soil except roots (10% of sites) and applying a fixed Rh-soil:Rsoil ratio (10% of sites) (see Methods for details). All these approaches have methodological uncertainties^26^. However, the fact that the difference between NEP_BM_ and NEP_EC_ was not affected by the four approaches to measure Rh-soil (Table 3) and that all four approaches presented an underestimation of NEP_BM_ very similar to the global difference between NEP_BM_ and NEP_EC_ (Supplementary Fig. 2), point towards the conclusion that the low convergence between NEP_BM_ and NEP_EC_ is more likely to be related to underestimation of NPP than overestimation of Rh-soil. Underestimation of NPP is indeed considered as a key potential source of bias in the BM approach to estimate NEP (see Introduction). An analysis of the primary factors possibly causing NPP underestimation based on the literature indicated that, on average, our NPP values might be underestimated by roughly 20% because incomplete assessment of mycorrhiza NPP and NPP related to branch turnover, which were taken into account in only very few of the sites (see Methods). Furthermore, additional underestimation could have been caused by minor NPP components typically neglected (for example, root exudation, net accumulation of non-structural carbohydrates, herbivory consumption, production of volatile organic compounds^22^), which in general contribute between 1 and 4% of NPP^16^,^27^,^28^,^29^. The low convergence between NEP_EC_ and NEP_BM_, when NEP_BM_ was obtained from NPP and Rh, and the difficulty in quantifying NPP, suggests that stock change approaches may be a valid BM alternative to quantify NEP. For example, even if this analysis was less robust because of the small sample size, we discovered that when NEP_BM_ was based on repeated stock inventories, the difference between EC- and BM-based estimates of NEP was not statistically significant.

Except for Reco in the boreal zone (as mentioned above), estimates of Reco and GPP did not show significant differences between methods. This high methodological convergence increases the confidence in the knowledge gained using only EC^10^,^11^ or BM^12^,^13^ and favours the integration of EC- and BM-based estimates in synthesis studies, model-data fusion and model development. The higher EC–BM convergence for Reco and GPP than NEP might appear counter-intuitive at first, as Reco and GPP are derived from NEP (for EC) or rely on the measurements of the same component processes of NEP (for BM). However, the different degree of EC–BM convergence among the carbon fluxes is coherent with our results and likely related to the small magnitude of NEP and to the partial offset of errors of opposite direction for Reco and GPP. On the one hand, the magnitude of NEP fluxes is only about 15–20% of the magnitude of Reco and GPP. Therefore, NEP estimates can be more susceptible than Reco and GPP to methodological inaccuracies. On the other hand, it is important to realize that high convergence of Reco and GPP is not definitive proof of lack of systematic biases, but could be caused by error compensation. Three examples can be provided. For GPP_BM_, the underestimation of NPP (discussed above) might be compensated for by overestimation of autotrophic respiratory fluxes, such as Rleaf (Fig. 3b,c). For Reco_BM_, overestimation of Rleaf may be compensated for by an underestimation of Rsoil (see above). For Reco_EC_, the overestimation caused by overlooking the light inhibition of Rleaf, might be offset by missing CO_2_ fluxes. Overall, the compensating aspects of Reco and GPP are not important for landscape- and large-scale assessment of ecosystem CO_2_ fluxes but should nonetheless be investigated in depth at site-level so that experimentalists can better evaluate the measurement strategy and modelers have the appropriate information on key ecosystem C cycle processes.

In conclusion, our study has shown four key findings. First, forest NEP estimates obtained with BM and EC differ significantly, particularly for the boreal zone, where the source/sink status is sensitive to the measurement technique. Second, BM and EC-based estimates of Reco and GPP are globally comparable for forest ecosystems but error compensation is likely to play a role in the convergence. Third, there are indirect but multiple indications that the potential biases associated with EC are, in aggregate, sufficiently amended by post-processing of the data. Fourth, BM approaches underestimating NPP and neglecting light inhibition of leaf dark respiration show less convergence with EC estimates. Our results also provide manifold information with important practical implications, as they identify in which situations forest C fluxes can be measured with both EC and BM methodologies (with the choice of the method depending on factors such as scientific objectives, logistics, local researcher capacity and availability of labour) and it is safe to integrate EC- and BM-based C flux estimates in synthesis studies or model-data fusion (Table 4). On the other hand, we report the mean values of difference against which the discrepancy between EC and BM can be tested in cases of low convergence (for example, for boreal forests) and which methodological aspects need to be taken most urgently into account to improve the convergence between EC and BM. Finally, we make also available the uniform forest C cycle data set that we compiled using quality-checked EC and BM data. All these evidences, elucidations and tools will substantially improve our ability to assess and simulate the CO_2_ exchange of terrestrial ecosystems.

## Methods

### Overview study data set

We constructed a data set of NEP, Reco and GPP obtained from BM and EC methodologies. The data set contains annual flux estimates, uncertainties, key information about the measurement techniques and ancillary data on environmental and stand variables. Data were retrieved from ISI-Web literature and existing databases such as the global database of forest C cycle^25^, FLUXNET^30^ and the European Flux Database Cluster^31^.

EC data are consistent as calculated through standard procedures, quality-check and data processing^5^. However, the NEP partitioning methods used to produce Reco and GPP can vary substantially and we classified them into three categories: methods based on night time data following Reichstein *et al*.^10^ or very similar algorithms^32^,^33^,^34^, methods based on daytime data following Lasslop *et al*.^9^ and calculation of Reco from sundown respiration following van Gorsel *et al*.^35^. As the latter method was only applied at two sites, the analyses on the partitioning method were actually focused on the night time and daytime methods only. Contrary to EC, no standard procedures exist for BM and data in the literature are highly heterogeneous, reflecting their diverse scope (for example, assessing the impact of a manipulative experiment, stand level estimates, regional assessments), local researcher capacity and practical reasons (for example, logistics, availability of labour). To obtain a uniform and quality-checked BM data set, we performed three operations. First, we retrieved details on BM for each site and classified them in multiple categories of methodological approach and up-scaling methods. Second, we considered in the analysis only sites fulfilling a pre-defined set of data quality criteria. Third, we related the data uncertainty to the technique adopted. Full details about the BM data set and its construction are reported below in four sections: annual estimates of production and respiration in BM data set, variables of BM data set for estimation of flux uncertainty, methodological variants of BM approach, and NEP biometric data from stock inventories.

The key environmental and stand variables used in the analysis are two indices of topographical complexity (that is, elevation variability and topographical slope) and LAI, which are thought to be related to bias in the EC-based estimates of C fluxes (see Introduction). The indices of topographical complexity were measured within a 2,430 × 2,430 m quadrat, centered at the EC tower, obtained from NASA ASTER DEM data^36^. The elevation variability was the standard deviation of the elevation of 729 pixels composing the quadrat. The topographical slope was derived from the elevation and distance of the highest and lowest pixels within the quadrat. The LAI was derived from the literature. The other variables tested for possible systematic influences on EC and BM include leaf characteristics, climatic features and site fertility. Leaf characteristics refer to leaf habit (evergreen, deciduous or mixed) and leaf type (needleleaved, broadleaved or mixed), which might affect the measurement of LAI^21^ and, consequently, the upscaling of leaf measurements to the stand level. Climatic features comprise climate zone (boreal, temperate or tropical), mean annual temperature and mean annual precipitation, which might affect both the instrument performance and the post-processing of the data (which involve different type of modelling and extrapolations)^5^,^37^. Site fertility was considered because it was recently found as the key driver of the CO_2_ exchange in forests^38^. It was classified in three categories (infertile, moderately fertile or fertile) based on soil type, physicochemical soil properties, and human amendments/degradation^29^,^38^,^39^. All the environmental and stand variables were derived from the literature or other databases and are reported in Supplementary Table 4 and Supplementary Data 1.

### Annual estimates of production and respiration in BM data set

The main component processes of the ecosystem C cycle considered are: NPP, aboveground Ra, Rsoil and its components (root autotrophic respiration, Rroot and Rh-soil) and Rh-cwd:


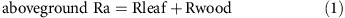



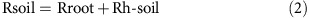


These variables are combined to obtain NEP, Reco and GPP:


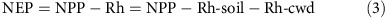



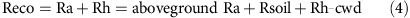



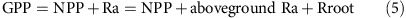


A full list of the variables (for example, NPP, Ra) for each ecosystem component (for example, foliage, wood, fine roots, soil) is reported in Supplementary Table 7. The methods used to measure these variables are briefly reported below, whereas extended methodological information for each site (for example, set-ups and measuring protocols, methods used to integrate point measurements to annual scale, methods to scale up tree level data to the stand level) is reported in Supplementary Methods. For sites with multiple-years data of NEP, Reco and GPP but with a combination of direct and indirect measurements, only years with direct measurements were considered.

Net primary production. Sites were selected when at least the three major components of the ecosystem production were measured: wood NPP, foliage NPP and fine root NPP. Wood NPP (stem, branches and coarse roots) was obtained as the increment in wood standing biomass from consecutive tree size (typically diameter) surveys and allometric relationships between tree size and wood standing biomass. Detailed information about the allometric relationships used at each site is reported in Supplementary Table 8. The quality of the relationships was classified into three categories according to their degree of species-specificity (species-specific versus generic), their geographical origin (site-specific versus regional) and their degree of flexibility (full dependency on tree size versus partial use of fixed productivity ratios). The categories are: high quality, for species- and site-specific relationships without fixed productivity ratios; moderately quality, for species-specific relationships without fixed productivity ratios but not site-specific, and low quality, for generic and/or not site-specific relationships employing also fixed productivity ratios. Foliage NPP was typically obtained from leaf litter collected with litter traps (for both deciduous and evergreen species) or from tree size surveys and allometric relationships between tree size and current-year leaf biomass (for evergreen species). Fine root NPP was measured with different methods, which can be classified into three main categories: sequential coring^40^ (for 26% of our sites), minirhizotron-based technique^41^ (23% of sites) and ingrowth cores^42^ (19% of sites). In addition, we aggregated the other techniques (for example, process-based modelling^43^, empirical modelling^44^, mass balance approach^45^) in a fourth category named other methods. For a comparison across methods see Milchunas^23^. Only sites with site-specific estimates of wood, foliage and fine root NPP were included. Therefore, sites with fine root NPP derived from aboveground NPP, generic algorithms or global models were not considered. The majority of the sites (70%) meeting the requirement on wood, foliage and fine root NPP also presented understory NPP (generally measured with a combination of allometric relationships between plant dimension and biomass and/or harvest techniques^17^). NPP due to branch turnover (from branch fall surveys), reproductive organs (from litter traps) and herbivory (from leaf area or biomass consumption) was considered in 30–40% of the sites. NPP due to production of volatile organic compounds and mycorrhizae production was considered in only 10% of the sites.

Aboveground respiration. To be included in our quality-checked BM data set, sites needed to comprise at least site-specific estimates of Rleaf and Rwood, fully independent to EC data. Rleaf was typically measured with chambers and infrared gas analyser, *in situ*, on canopy leaves^37^,^46^, or *in vitro*, on leaves of freshly cut branches^42^,^47^. Rwood was always measured with chambers and infrared gas analyser. Measurements were typically performed during various occasions in the growing and dormant season and integrated at annual level by using empirical models that related respiration to temperature, water status or other environmental variables^37^. Data were scaled up at stand level using LAI or leaf biomass, for Rleaf, and sapwood volume or area, for Rwood. About 60% of the sites presented also measurements of understory respiration (Ru; absence of Ru data was however not considered as a criterion for excluding the site). Measurement methods of Ru were similar to the ones for Rleaf and Rwood, and, often, Ru data were not presented separately but included into Rleaf and Rwood.

Belowground respiration. Rsoil was needed for a site to be included in our Reco_BM_ data set, whereas partitioning into Rroot and Rh-soil was needed for inclusion in the data set of GPP_BM_ and NEP_BM_, respectively (Supplementary Table 7). Rsoil was measured by using various soil chamber systems which fall into three major categories: closed static chambers or NSNF, closed dynamic chambers or NSF, and open dynamic chambers or steady-state through-flow chamber^19^. However, because the latter system was only used in two of our sites, we did not consider this system in our statistical analysis. On the other hand, for the NSF technique, we further classified the sites into two groups, according to whether the system prescribed scrubbing of CO_2_ before the flux measurements (for example, Li-Cor LI-6400-09 system; LI-COR Biosciences, Lincoln, USA) or not^19^. Measurements not performed continuously were integrated at annual scale using empirical models relating soil respiration to temperature and/or soil water status. Various methods were applied to partition Rsoil into Rroot and Rh-soil. We grouped them into four categories: root exclusion methods (used in 57% of the sites), that directly measure Rh-soil *in situ* and indirectly derive Rroot from equation (2); estimation of Rroot (20% of sites), both *in situ* (using root chambers), *in vitro* (using excised roots) or using models (process-based or empirical models) and indirectly deriving Rh-soil from equation 2; component integration (10% of sites), that prescribes the estimation of Rh-soil from the respiration of all components of the soil (for example, litter, mineral soil layers and so on) separately, often *in vitro* in the laboratory, and, as for the exclusion methods, indirectly estimates Rroot (equation (2)), and other methods, which mainly assume a fixed ratio between Rroot and Rh-soil^26^. See Hanson *et al*.^26^ for more details on all methods to partition Rsoil into Rroot and Rh-soil.

Other respiratory components. Decomposition of coarse woody debris (Rh-cwd) can be a significant part of the total ecosystem respiration. Rh-cwd was therefore considered when reported (for 65% of the sites) but sites missing it were not excluded. Rh-cwd was typically derived from surveys of standing dead wood and estimates of decays rates, or, in a minority of cases, from chamber measurements of CO_2_ exchange or wood decomposition from successive surveys.

### Variables of BM data set for estimation of flux uncertainty

Data uncertainty of NEP, Reco and GPP was calculated following the method described below. The data required for the estimation of the flux uncertainty are: the site latitude, the number of replicated years of measurement, and a general label of data quality. The latter has two levels: high quality data, for sites with only site-specific measurements, or fair quality data, for sites with measurements comprising also not site-specific relationships and/or models.

### Methodological variants of BM approach

BM data were often obtained at different sites with different BM techniques or data processing. Therefore, we reported in the BM data set the employed approach for each site and measured variable. This was done to analyse the impact of the BM methodology on the convergence between EC and BM fluxes. In particular, we considered 17 methodological variants of the BM technique (Table 3). For this analysis, we paid more attention to variables that had a large numerical impact on the stand C cycle (for example, Rsoil, fine root NPP, Rleaf, Rh-soil), whereas we paid less attention to variables that had a small numerical impact (for example, Rwood, Rh-cwd).

### NEP biometric data from stock inventories

NEP_BM_ is not only obtainable from measurements of production and respiration (equation (3)) but also derivable from the difference in ecosystem C stocks between two points in time^12^. NEP_BM_ estimates obtained with the latter approach are indicated here as NEP_BM-ΔS_. Ideally, C stocks should comprise vegetation, necromass and soil, with correction for lateral C losses (for example, harvests, leaching of dissolved organic carbon)^48^. In practice, complete C stock inventories are seldom done. Therefore, we included in the NEP_BM-ΔS_ data set all sites in the literature (*n*=7) with the repeated assessment of at least the two major ecosystem C stocks that is, wood and soil.

### Data uncertainty

As flux uncertainty was available only for a minority of sites (and its calculation method was highly inconsistent among studies), the uncertainties in fluxes were approximated uniformly following Luyssaert *et al*.^25^ These authors proposed that the flux uncertainty for a site (*s*) can be approximated by considering three elements. First, the typical range of the flux value for the site biome (*p*) set as the maximal potential uncertainty range. Second, a reduction factor (RF), depending on the measuring methodology, that reduces the maximal potential uncertainty range (for example, a precise method would reduce the uncertainty interval more than an imprecise method). Third, the number of measurement years (*l*), with more replicate-years reducing the uncertainty interval. Thus:


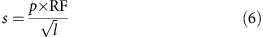


For example, to determine the NEP_EC_ uncertainty of a temperate forest with two years of measurements, the Luyssaert *et al*.'s approach considers that NEP of temperate forests typically ranges between −100 and 600 gC m^−2^ y^−1^ (thus s=*p*=±350 gC m^−2^ y^−1^), that EC is precise and reduces the uncertainty to 30% of the baseline value (RF=0.3, thus *s*=±105 gC m^−2^ y^−1^) and that the presence of two measurement years further reduces the uncertainty to *s*=±74 gC m^−2^ y^−1^. In general, according to this method, *p* of NEP is assumed to be 350 gC m^−2^ y^−1^ for extratropical forests and 700 gC m^−2^ y^−1^ for tropical forests^25^, *p* of Reco and GPP depends on latitude and varies from 500 to 1000, gC m^−2^ y^−1^ (ref. 25), and that *RF* of BM is 0.3, as for EC, for sites with high quality data and 0.6 for sites with fair quality data (see above).

The adopted approach produces uncertainty intervals comparable to the directly estimated uncertainty for EC^25^. Here, we observed that good agreement was achieved also for the BM fluxes of extratropical forests but that relevant mismatches were detected for Reco and GPP of tropical sites where the Luyssaert *et al*.'s approach underestimated the measured uncertainty by 60–70% (Supplementary Table 9). We considered this important discrepancy not crucial for our analysis because we had only four tropical sites and repetition of the analysis with corrected uncertainty values of tropical sites (to match the directly estimated uncertainty) did not change the main results (data not shown).

### Data analysis

The data were analysed in four steps, which are described below. For each case, note that the comparison of the EC and BM fluxes was performed for sites with data referring to the same vegetation cover (or footprint) and to the same period or with minor temporal mismatches (for example, BM fluxes available for a three-year period and EC fluxes for a 2-year period) generally considered suitable for the EC and BM comparison by the original investigators (for 74% of the sites the BM and EC data were from published EC–BM comparison at the site-level).

Agreement between EC and BM. We obtained a first indication of the agreement between EC- and BM-data by calculating the regression of BM versus EC estimates along the entire range of flux measurements. Major-axis regressions were used because both variables had error terms of similar magnitude^49^. Agreement was inferred from the slope of the regression line and the correlation indicated by *R*^2^. In addition, for each flux and climatic zone, the agreement between the EC and BM estimates was more stringently tested by a paired *t*-test (in case the differences between pairs were normally distributed according to Shapiro–Wilks' test) or Wilcoxon signed-rank test (in case the differences between pairs were not normally distributed). The same approach was used to compare NEP_EC_ and NEP_BM-ΔS_ (see above).

Impact of environmental and stand variables on the EC and BM convergence. We tested whether the difference between the EC- and BM-based estimates was systematically related to elevation variability and topographical slope (indices of topographical complexity), LAI, leaf habit and leaf type (canopy characteristics), climate zone, mean annual temperature and mean annual precipitation (climate variables) and site fertility (see above). The tests comprised univariate analyses performed regressing (with an ordinary least squares regression) the difference between the EC- and BM-based estimates and each variable, separately. To fully exploit the information available for each site, the impact of flux data uncertainty was added to the analysis by using the inverse of the flux uncertainty as weighing factor among sites (thus giving lower weight to sites with higher uncertainty on the estimates). Each analysis met the normality of residuals (tested with Shapiro-Wilks' test) and the assumption of homoskedasticity (tested with Breusch-Pagan test), except in a few cases for which the White method (for heteroskedasticity correction) was used instead^50^.

Impact of different methodological variants on the convergence between EC and BM. The impact of different methodological approaches on the convergence between EC and BM-based estimates was tested as above with ordinary least squares regressions weighted by the inverse of the flux uncertainty. These univariate analyses served well for the purpose of the study, as preliminary analyses showed typically no relevant two-term interactions. The few significant (*P*<0.05) two-terms interactions (1 out 55 cases for GPP, 5 out of 66 for Reco and 1 out of 6 for NEP) did not have logical meaning but were related to the small sample size, that is, singularities. The only exception was represented by a significant interaction between soil respiration chamber type and accounting for light inhibition of leaf respiration for Reco, which was considered in the data analysis (see Results). For BM, the methodological variants were 17 (Table 3). For EC, the only methodological variant was the NEP partitioning method which was of two types (see above).

Analysis of NPP data. NPP is a key component for the determination of NEP_BM_ and GPP_BM_. Five methodological flaws can typically have a significant (>10%) and unidirectional (underestimation) impact on NPP estimates in forests^22^: not accounting tree mortality, assuming life span of fine roots to be 1 year, coarse measurements of leaf NPP in tropical forests, not measuring mycorrhizal NPP and rhizodeposition, and not correcting for NPP related to branch turnover. The first three cases are less relevant in our data set as: site tree mortality was normally assessed, fine root production was estimated for each site and tropical sites are few in our data set and their leaf NPP was quantified accurately. On the other hand, we detected than only three and six sites (out of 31) took into account mycorrhizal NPP and NPP related to branch turnover, respectively. The impact of these missing terms was estimated by gap-filling the original NPP values using the average values of branch turnover related NPP from our data set (which was equivalent to 22% of aboveground wood NPP or 8% of total NPP, *n*=6; Supplementary Table 2) and of mycorrhizal NPP from the literature (which was equivalent to 14% of total NPP, *n*=6, and in agreement with culture studies^51^; Supplementary Table 10). The gap-filled NPP estimates were on average 20% larger than the original NPP estimates.

Overall, the analyses were performed for all fluxes, that is, NEP (NEP_BM_ versus NEP_EC_), Reco (Reco_BM_ versus Reco_EC_) and GPP (GPP_BM_ versus GPP_EC_). For Reco and GPP, to ensure that the impact of each given site was independent on the flux magnitude, the analyses were performed using the relative difference between the EC- and BM-based flux estimates (for example, for GPP: (GPP_BM_ – GPP_EC_)/((GPP_EC_ + GPP_BM_)/2). For NEP, the latter approach was impeded by the presence of both positive and negative values. We intentionally retained outliers in our analyses because they could represent cases with high discrepancy between the methodologies and thus of relevance for our scope. All analyses were performed within the R platform^52^.

### Data availability

The data that support the findings of this study are included in Supplementary Data 1. Data are from the literature and public databases. Details about the data and the data sources are reported in Supplementary Tables 1-5.

## Additional information

**How to cite this article:** Campioli, M. *et al*. Evaluating the convergence between eddy-covariance and biometric methods for assessing carbon budgets of forests. *Nat. Commun.* **7,** 13717 doi: 10.1038/ncomms13717 (2016).

**Publisher's note**: Springer Nature remains neutral with regard to jurisdictional claims in published maps and institutional affiliations.

## Supplementary Material

Supplementary Information Supplementary Figures 1-4, Supplementary Tables 1-10, Supplementary Methods and Supplementary References.

Supplementary Data 1 This file contains the key dataset used in the analysis.

## Figures and Tables

**Figure 1:**
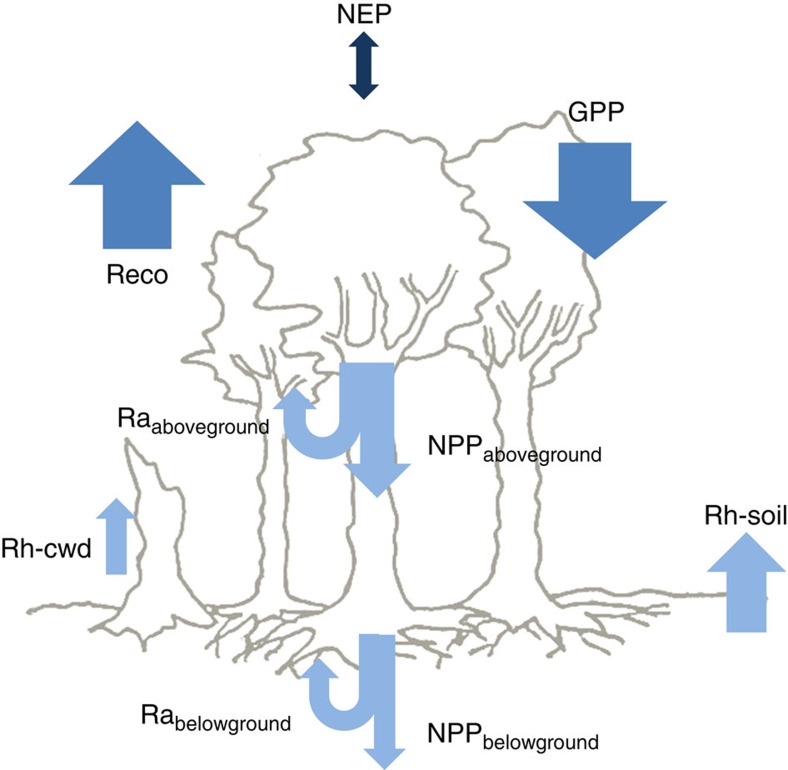
Schematic representation of the major components of the forest carbon cycle. Ra_aboveground_ and Ra_belowground_: above- and belowground autotrophic respiration, respectively (their sum is indicated as Ra); Rh-soil and Rh-cwd: heterotrophic respiration from soil and coarse woody debris, respectively (their sum is indicated as Rh); NPP_aboveground_ and NPP_belowground_: above- and belowground net primary production, respectively (their sum is indicated as NPP); Reco: ecosystem respiration (Reco=Ra+Rh); GPP: gross primary production (GPP=NPP+Ra), and NEP: net ecosystem production (NEP=GPP−Reco=NPP−Rh). Each flux is associated with an arrow. Arrows pointing down indicate carbon (C) uptake, arrows pointing up indicate C release, whereas the up-down arrow indicates that both C release and C uptake can occur. The dark blue arrow indicates NEP, the mid-blue arrows indicate the primary components of NEP (Reco and GPP), whereas the light blue arrows indicate the components of Reco and GPP.

**Table 1:** Positive and negative characteristics of eddy-covariance and biometric methods.

**Characteristics of the technique**	**EC**	**BM**
Directness of approach^1^,^25^	++* /−−†	+
Temporal and spatial up-scaling^1^,^18^	+	−−
Applicability to small-footprint studies	−	++
Interference with sampled system^1^	++	−
Sensitivity to low turbulence environment^1^,^8^,^18^	−−	+
Impact of measuring set-up on microclimate^1^,^53^	+	−
Impact of complex terrain^1^	−	++
Compartment-level understanding and partitioning of fluxes and allocation^16^	−	++
Unaccounted/miscounted carbon fluxes at tree organ/ecosystem compartment level (e.g. understory fluxes, herbivory)^16^,^54^,^55^	++	−
Set-up costs^56^	−−	++
Ongoing labour requirements^1^	+	−
Technical capacity requirements for data collection and processing	−−	+

Very positive (++), positive (+), negative (−) or very negative (−−) characteristics of the eddy-covariance (EC) and biometric (BM) methods used for the determination of net ecosystem production (NEP), ecosystem respiration (Reco) and gross primary production (GPP) of forest ecosystems.

^*^NEP.

^†^Reco and GPP.

**Table 2:** Comparison of carbon fluxes obtained from eddy-covariance or biometric methods for forests worldwide and in the main climatic zones.

	**BM versus EC**			**BM**_**ΔS**_ **versus EC**
	**NEP**	**Reco**	**GPP**	**NEP**
*Global*				
Site replicates (*n*)	31	25	18	7
Absolute difference (mean±s.e.m)	−98±32	120±61	25±67	32±87
Significance difference (*P*)	0.0042**	0.061^+^	0.71	0.73
Relative difference (mean±s.e.m in %)	NA	13±4	5±4	NA
				
*Boreal*				
Site replicates (*n*)	6	6	4	1
Absolute difference (mean±s.e.m)	−167±44	189±75	89±59	26
Significance difference (*P*)	0.013*	0.031*	0.23	NA
Relative difference (mean±s.e.m in %)	NA	18±7	8±5	NA
				
*Temperate*				
Site replicates (*n*)	22	15	11	6
Absolute difference (mean±s.e.m)	−95±28	160±85	59±100	33±102
Significance difference (*P*)	0.0028**	0.079^+^	0.57	0.76
Relative difference (mean±s.e.m in %)	NA	16±6	6±6	NA
				
*Tropical*				
Site replicates (*n*)	3	4	3	NA
Absolute difference (mean±s.e.m)	10±275	−137±138	−182±119	NA
Significance difference (*P*)	1.0	0.39	0.26	NA
Relative difference (mean±s.e.m in %)	NA	−5±5	−5±3	NA

Statistics of the comparison of net ecosystem production (NEP), ecosystem respiration (Reco) and gross primary production (GPP) at global scale and for the boreal, temperate and tropical zones, separately, assessed with eddy-covariance (EC) and two types of biometric methods: standard biometric methods based on measurements of production and respiration (BM) and biometric methods based on consecutive inventories of ecosystem carbon stocks (BM_ΔS_). The difference between methods is expressed as Absolute difference (BM estimate − EC estimate) and Relative difference (BM estimate − EC estimate)/((BM estimate + EC estimate)/2). Difference at 0.001<*P*<0.01, 0.01<*P*<0.05 and 0.05<*P*<0.10 are marked with **, * and +, respectively. The notation NA indicates data not available.

**Table 3:** Relationship between the difference in forest carbon fluxes estimated from eddy-covariance and biometric methods and site or methodological characteristics.

**Variables**	**Category (units)**	**NEP**_**EC**_**–NEP**_**BM**_		**Reco**_**EC**_**–Reco**_**BM**_		**GPP**_**EC**_**–GPP**_**BM**_	
		**Difference**		**Relative difference**		**Relative difference**	
		***P***	***R***^**2**^	***P***	***R***^**2**^	***P***	***R***^**2**^
*Topography, environmental and stand variables*							
Elevation variability	m	0.89	<0.01	0.82	<0.01	0.13	0.13
Topographical slope	%	0.80	<0.01	0.42	0.03	0.086†	0.17
Leaf area index	m^2^ m^−2^	0.36	0.03	0.91	<0.01	0.60	0.02
Leaf type	Needleleaved/broadleaved/mixed	0.27	0.04	0.24	0.07	0.44	0.04
Leaf habit	Evergreen/deciduous/mixed	0.47	0.02	0.32	0.06	0.11	0.16
Fertility	Low/medium/high	0.28	0.05	0.61	0.01	0.57	0.07†
Climate zone	Boreal/temperate/tropical	0.16	0.07†	0.12	0.15	0.34	0.08
Mean annual precipitation	mm per year	0.31	0.02†	0.25	0.06	0.34	0.06
Mean annual temperature	°C	0.42	0.05†	0.13	0.10	0.22	0.09
							
*Methodological variants*							
Methods to measure fine root NPP	Sequential coring/ingrowth cores/minirhizotron technique/other	0.22	0.11	NA	NA	0.11	0.32
Allometric relationships to measure wood NPP	Low/moderate/high quality	0.58	0.04	NA	NA	0.69	0.02
Method of measuring leaf NPP‡	leaf fall collection/allometry	0.43	0.04	NA	NA	0.85	<0.01
Chamber method to measure Rsoil	NSNF/NSF	NA	NA	0.008**	0.34	NA	NA
Scrubbing of CO_2_ before Rsoil measurement§	Yes/no	NA	NA	0.22	0.09	NA	NA
Methods to measure Rh-soil	Root exclusion/indirectly from estimation of root respiration/component integration/other	0.59	0.03	NA	NA	NA	NA
Consideration of Rh-cwd	Yes/no	0.16	0.07	0.84	<0.01	NA	NA
Variables of models for integration of Rsoil at annual scale	Soil temperature/soil temperature and water	NA	NA	0.16	0.10	NA	NA
Variables of models for integration of Rleaf at annual scale	Temperature/temperature in combination with other	NA	NA	0.13	0.11	0.32	0.07
Parameterization of models for integration of Rleaf at annual scale	Site-specific/generic	NA	NA	0.043*	0.17	0.40	0.04
Variability of temperature sensitivity of Rleaf in models for integration of Rleaf at annual scale	Yes/no	NA	NA	0.23	0.06	0.65	0.013
Consideration of light inhibition of leaf dark respiration in Rleaf	Yes/no	NA	NA	0.041*	0.17	0.43	0.04
Consideration of leaf growth respiration in Rleaf	Yes/no	NA	NA	0.94	<0.01	0.64	0.14
Consideration of wood growth respiration in Rwood	Yes/no	NA	NA	0.34	0.039	0.54	0.024
Variables of models for integration of Rwood at annual scale	Temperature/temperature in combination with other	NA	NA	0.25	0.07	0.086†	0.21
Variable used to scale up Rwood at stand level	Wood volume/wood area	NA	NA	0.37	0.04	0.44	0.04
Separation contribution of branch and stem in Rwood	Yes/no	NA	NA	0.68	<0.01	0.42	0.04

NA, not applicable; NPP, net primary production; NSF, non-steady-state through-flow chamber (closed dynamic chamber); NSNF, non-steady-state non-through-flow chamber (closed static chamber); Rleaf, leaf respiration; Rh-cwd, heterotrophic respiration of coarse woody debris; Rh-soil, heterotrophic soil respiration; Rsoil, soil respiration; Rwood, aboveground wood respiration.

Statistics (significance level (*p*) and *R*^2^) for the ordinary least squares regressions between the difference in estimates of net ecosystem production (NEP), ecosystem respiration (Reco) and gross primary production (GPP) determined with eddy-covariance (subscript EC) or biometric methods (subscript BM) and site characteristics or methodological variants of biometric methods. Difference at 0.001<*P*<0.01, 0.01<*P*<0.05 and 0.05<*P*<0.10 are marked with **, * and +, respectively.

^†^In case of heteroskedasticity the square of Pearson's correlation was reported.

^‡^Only for sites dominated by evergreen species.

^§^Only for sites with NSF system to measure Rsoil.

**Table 4:** Risk of lack of convergence between estimates of forest carbon fluxes obtained from eddy-covariance and biometric methods according to site and methodological characteristics.

**Flux**	**Variables**		
*Overall*			
NEP	High		
Reco	Moderate		
GPP	Low		
			
*Climate zone*			
	*Boreal*	*Temperate*	*Tropical*
NEP	High	High	Low
Reco	High	Moderate	Low
GPP	Low	Low	Low
			
*Canopy features*			
	Leaf type*	*Leaf habit*†	*Leaf area index*
NEP	Low	Low	Low
Reco	Low	Low	Low
GPP	Low	Low	Low
			
*Topography and soil characteristics*			
	*Altitude variability*	*Slope*	*Fertility*
NEP	Low	Low	Low
Reco	Low	Low	Low
GPP	Low	Moderate	Low
			
*Compartment measured with biometric methods*			
	*Leaves*	*Wood*	*Soil*
NEP	Low	Moderate‡	Moderate§
Reco	High||	Low	High¶
GPP	Moderate||	Moderate‡	Moderate§

The risk of lack of convergence between eddy-covariance (EC) and biometric methods (BM) estimates of net ecosystem production (NEP), ecosystem respiration (Reco) and gross primary production (GPP) for forests is reported according to climatic zone, canopy features, site (topography and soil) characteristics, and the main ecosystem compartments measured with BM, and expressed in three levels: low (non-significant difference between BM and EC estimates and/or lack of systematic biases), moderate (difference between the BM and EC estimates at 0.05<*P*<0.10 and/or potential of systematic biases) and high (significant difference at *P*<0.05 between the BM and EC estimates and/or systematic biases).

^*^Needleleaved, broadleaved or mixed.

^†^Evergreen, deciduous or mixed.

^‡^Not considering branch turnover in estimates of net primary production (but with an adequate assessment of the other components of the wood production, see Methods).

^§^Not considering mycorrhizal production (but with an adequate assessment of root production, see Methods).

^||^Not considering light inhibition of leaf dark respiration.

^¶^Use of lower quality chamber system to measure soil respiration.
