# Supplementary material for: Evaluating the convergence between eddy-covariance and biometric methods for assessing carbon budgets of forests
Source: Nat Commun. 2016 Dec 14;7:13717. doi: 10.1038/ncomms13717 (PMC5171944; doi:10.1038/ncomms13717)
Supplement: Supplementary Information — Supplementary Figures 1-4, Supplementary Tables 1-10, Supplementary Methods and Supplementary References. [file ncomms13717-s1.pdf]

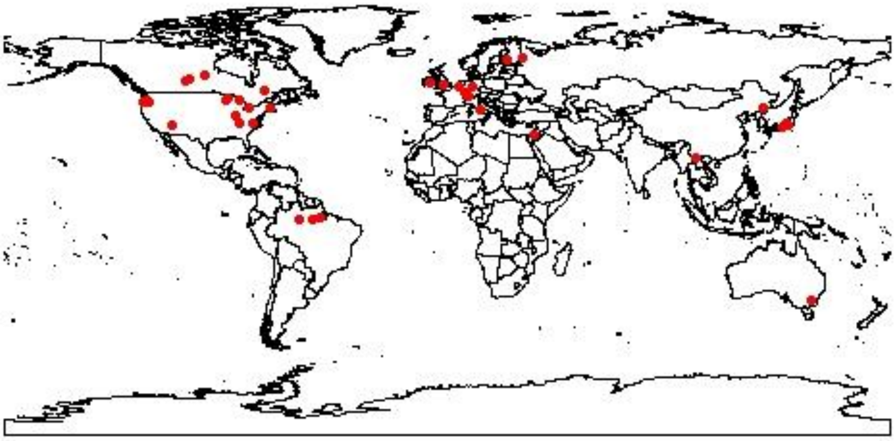

Supplementary Figure 1. Geographical location of the study sites. The map was produced using the freely available package rworldmap of the R platform[1], for which the GNU General Public License applies[2]

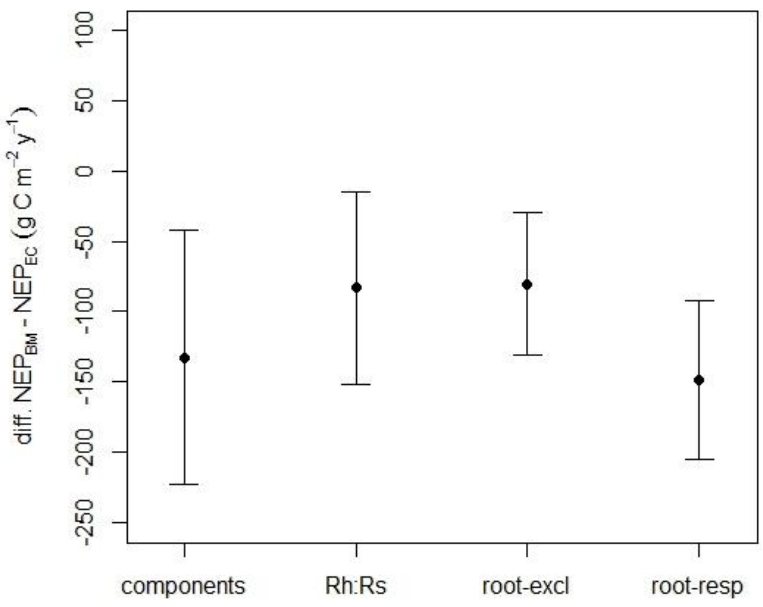

Supplementary Figure 2. Difference of net ecosystem production (NEP) from biometric methods (BM) and eddy-covariance (EC) according to the different method used to measure soil heterotrophic respiration in BM: (i) components integration (components), (ii) fixed ratio between soil heterotrophic respiration and total soil respiration (Rh:Rsoil), (iii) root exclusion method (root-excl), and (iv) measurements of soil respiration and root respiration (root-resp) (see Methods for more details on the four techniques). Points: mean; bars: s.e.m.

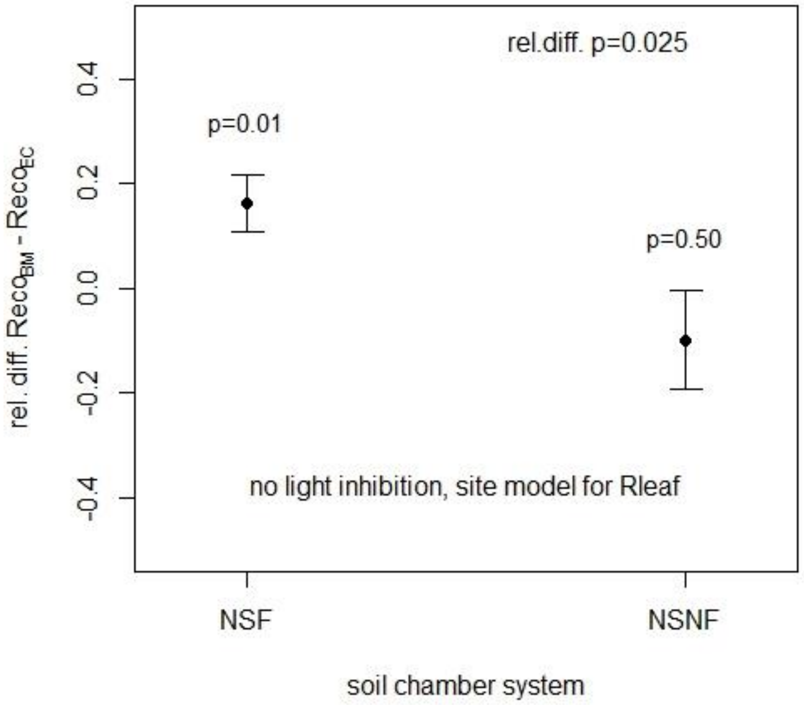

Supplementary Figure 3. The relative difference between ecosystem respiration from biometric methods ($Reco_{BM}$) or eddy-covariance ($Reco_{EC}$) [($Reco_{BM}$ – $Reco_{EC}$)/(($Reco_{EC}$ + $Reco_{BM}$)/2)] when using different chamber systems to measure soil respiration (NSNF: non-steady-state non-through-flow chamber, NSF: non-steady-state through-flow chamber) for sites not accounting for light inhibition in estimating leaf respiration (Rleaf) but using site-specific parameterization for the empirical models scaling up the point measurements of Rleaf at the annual scale. Points indicate means with bars indicating s.e.m; the p value above each point indicates the significance level of the difference between $Reco_{BM}$ and $Reco_{EC}$, whereas the significance level p of the effect of the chamber system is indicated as rel. diff. p (relative difference between $Reco_{BM}$ and $Reco_{EC}$) and is reported in the top right of the panel.

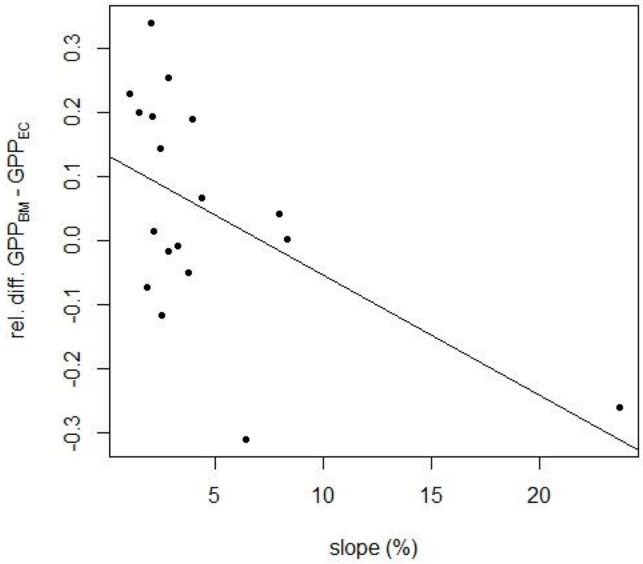

Supplementary Figure 4. Relationship between the relative difference between gross primary production from biometric methods (GPP_{BM}) and eddy-covariance (GPP_{EC}) [(GPP_{BM} – GPP_{EC})/((GPP_{EC} + GPP_{BM})/2))] and site slope.

Supplementary Table 1. Annual values of net ecosystem production (NEP, gC m^{-2} y^{-1}) from biometric methods (NEP_{BM}) and eddy-covariance (NEP_{EC}), ecosystem respiration (Reco, gC m^{-2} y^{-1}) from biometric methods ($Reco_{BM}$) and eddy-covariance ($Reco_{EC}$) and gross primary production (GPP, gC m^{-2} y^{-1}) from biometric methods (GPP_{BM}) and eddy-covariance (GPP_{EC}) of the study dataset.

site	Fluxnet	measurement period		NEP		Reco		GPP	
		BM	EC	NEP_{BM}	NEP_{EC}	$Reco_{BM}$	$Reco_{EC}$	GPP_{BM}	GPP_{EC}
Caxiuanã[3-5]	BR-Cax	2004-2011	1999	23	560	3205	3070	3228	3630
Changbai Mountains[6,7]	CN-Cha	2003	2003	NA	NA	1242	1292	NA	NA
Chibougamau EOBS[7-9]	CA-Qfo	2005	2005	-238	-15	1032	702	794	687
Collelongo[10,11]	IT-Col	2007	2007	NA	NA	764	727	NA	NA
Dooray[12]	IE-CLa	2003	2003	939	831	NA	NA	NA	NA
Duke Forest[13-15]	US-Dk3	1998	2004	502	523	1908	1733	2410	2256
Fujiyoshida[16,17]	JP-Fuj	1999-2008	2000-2008	302	388	NA	NA	NA	NA
Hainich[10,18,19]	DE-Hai	2000-2002	2002	260	564	NA	NA	NA	NA
Harvard[20]	US-Ha1	<1999	1999	165	200	NA	NA	NA	NA
Hesse[7,21-24]	FR-Hes	1997	1997	245	207	1199	1249	1444	1456
Huhus[25]	no	2001-2004	2001-2004	NA	NA	793	785	NA	NA
Lageren[26]	CH-Lae	<2009	2006-2009	306	435	NA	NA	NA	NA
Jacaranda[5,27]	BR-Ma2	2001	2000	NA	NA	3210	3180	NA	NA
Marys River Fir[28]	US-MRf	2011	2011	NA	NA	2009	1275	NA	NA
Metolius[29-31]	US-Me4	1996	1996	7	287	894	885	901	1172
Morgan Monroe[32]	US-MMS	1998-1999	1998-1999	325	262	NA	NA	NA	NA
NAU Centennial Thinned[33]	US-Fmf	2007	2007	-281	-51	NA	NA	NA	NA
NAU Centennial Undisturbed[33]	US-Fuf	2007	2007	-169	58	NA	NA	NA	NA
Prince Albert SSA SOJP[34,35]	CA-Ojp	1999-2000	2000	-20	78	NA	NA	NA	NA
Prince Albert SSA SOAS[36-40]	CA-Oas	1994-1995	1994	-151	206	1492	1117	1342	1323
SMEARII[7,41]	FI-Hyy	2003-2006	2003-2006	NA	NA	919	829	NA	NA
Sylvania hardwood[19,42-44]	US-Syv	2002-2003	2002-2003	44	102	1013	974	1057	1076
Takayama[45,46]	JP-Tak	1993-2003	1994-2003	210	237	NA	NA	NA	NA
Tapajos km 67[5]	BR-Sa1	1999-2006	2002-2005	219	-110	2770	3250	2989	3140
Thompson NSA NOBS[47-50]	CA-NS1	1994-2002	1999-2002	-132	15	NA	NA	NA	NA
Tumbarumba[7,51,52]	AU-Tum	2003	2003	434	517	1452	2069	1890	2586
TurkeyPointTP02[53,54]	CA-TP1	2006	2006	44	34	773	569	850	603
TurkeyPointTP89[7,53,54]	CA-TP2	2006	2006	482	727	1985	2055	2583	2782

TurkeyPointTP74[53,54]	CA-TP3	2006	2006	213	511	1278	751	1587	1262
TurkeyPointTP39[53,54]	CA-TP4	2006	2006	164	148	1526	1293	1762	1441
University of Michigan[55,56]	US-UMB	1999-2001	1999-2001	159	158	1449	1087	1608	1245
Walker Range[29,57,58]	US-WBW	1995-1996	1995-1996	260	523	1625	1036	1885	1559
Willow Creek[20,42,59,60]	US-WCr	<2002	2000-2003	146	262	1251	888	1397	1150
Wind River[61,62]	US-Wrc	1995-2000	1999-2000	35	130	NA	NA	NA	NA
Wytham Woods[63,64]	no	2008	2007-2008	170	130	2027	1980	2197	2110
Xishuangbanna[65]	CN-Xsh	2003-2006	2003-2006	358	119	2242	2475	2600	2594
Yamashiro[66]	JP-YMS	2000-2005	2000-2002	91	123	NA	NA	NA	NA
Yatir[10,67,68]	IL-Yat	2002	2002	NA	NA	731	456	NA	NA

Fluxnet: indicates if site is in Fluxnet (http://www.fluxdata.org/default.aspx) or *European Fluxes Database Cluster* (http://gaia.agraria.unitus.it/home/sites-list) with code. BM: biometric methods; EC: eddy-covariance; NA: not available.

Supplementary Table 2. Components and aggregated values of net primary production (NPP, gC m^{-2} y^{-1}) and heterotrophic ecosystem respiration (Rh, gC m^{-2} y^{-1}) determined for the study sites with biometric methods.

site	NPP												Rh	
	total	above[a]	below[b]	leaves	wood		roots		under.[e]	reprod.[f]	herbiv.[g]	other[h]	soil	cwd[i]
					incr.[c]	turn.[d]	coarse	fine						
Caxiuanã[4,5]	1377	922	423	368	382	106	55	368	NAs	42	24	32	1354	220
Chibougamau EOBS[9]	302	197	105	38	91	NAs	NAs	NAs	68	NA	NA	NA	540	NA
Dooray[12]	1266	NAs	NAs	NAs	NAs	171	NAs	43	NA	NA	NA	NA	318	9
Duke Forest[13,15]	707	NAs	NAs	NAs	NAs	NA	NAs	32	NAs	NAs	NAs	NAs	208	0
Fujiyoshida[17]	742	512	230	208	271	20	40	190	NA	33	NA	NA	420	20
Hainich[18,19]	697	NAs	NAs	NAs	177	NA	94	NAs	NAs	NAs	NA	NA	437	NA
Harvard[20]	565	320	245	130	130	NA	25	220	60	NAs	NA	NA	400	NA
Hesse[21-24]	643	510	133	131	379	NA	76	57	0	NA	NA	NA	338	60
Lageren[26]	761	651	110	242	369	NA	38	72	40	NAs	NA	NA	455	NA
Metolius[30,31]	228	136	92	59	77	NA	NAs	NAs	NAs	NA	NA	NA	221	NA
Morgan Monroe[32]	974	537	437	205	286	NA	24	413	18	14	14	NA	562	87
NAU Centennial Thinned[33]	240	132	108	50	70	NA	13	95	12	NA	NA	NA	509	12
NAU Centennial Undisturbed[33]	268	119	149	46	66	NA	11	138	7	NA	NA	NA	430	7
Prince Albert SSA SOJP[34]	170	90	80	20	70	NA	10	70	0	NA	NA	NA	170	20
Prince Albert SSA SOAS[37-40]	441	352	89	123	176	NA	32	57	53	NA	NA	NA	591	NA
Sylvania hardwood[19,43,44]	314	212	102	128	84	NA	3	99	0	NA	NA	NA	227	43
Takayama[45]	650	450	200	180	160	NA	20	180	110	NA	NA	NA	390	50
Tapajos km 67[5]	1499	1186	300	650	536	160	100	200	NA	NA	NA	13	830	450
Thompson NSA NOBS[47-49]	211	145	67	39	72	NA	9	57	35	NA	NA	NA	329	14
Tumbarumba[51]	1040	NAs	NAs	NAs	NAs	NA	NAs	NAs	NAs	NA	NA	NAs	606	NA

TurkeyPointTP02[53,54]	346	282	64	82	98	NAs	58	6	101	NA	1	NA	270	6
TurkeyPointTP89[53,54]	870	694	176	344	345	NAs	95	81	0	NA	4	NA	272	25
TurkeyPointTP74[53,54]	639	423	216	161	235	NAs	44	172	25	NA	2	NA	330	38
TurkeyPointTP39[53,54]	634	453	181	234	185	NAs	71	110	32	NA	2	NA	398	52
University of Michigan[55,56]	677	354	323	149	198	17	42	281	NAs	NAs	7	NA	518	NA
Walker Branch[57,58]	788	608	179	242	321	NA	16	164	7	16	22	NA	441	87
Willow Creek[20,59,60]	613	300	313	135	155	NA	31	282	10	NAs	NA	NA	502	79
Wind River[62]	597	449	142	135	233	NA	51	91	66	NAs	15	6	346	216
Wytham Woods[63]	704	442	262	240	165	NA	33	229	NA	37	NA	NA	531	3
Xishuangbanna[65]	880	NAs	NAs	NAs	NAs	NA	NAs	NAs	NAs	NAs	NA	NA	454	68
Yamashiro[66]	507	427	80	258	169	61	22	58	NAs	NA	NA	NA	366	50

(a) aboveground; (b) belowground; (c) NPP related to increment in standing wood biomass; (d) NPP related to branch turnover; (e) understory; (f) reproductive materials (e.g. seeds, fruits, inflorescences); (g) NPP lost because of herbivory; (h) NPP related to neglected NPP components (e.g. production of volatile organic compounds, mycorrhizal production, production of epiphytes, NPP related to dissolved organic carbon), and (i) heterotrophic respiration due to coarse woody debris. Green cells: data available; NAs: data not available separately but aggregated in total NPP or other NPP components (e.g. aboveground and belowground NPP); yellow cells and NA: data not available.

Supplementary Table 3. Components and aggregated values of ecosystem respiration (Reco, gC m^{-2} y^{-1}) determined for the study sites with biometric methods.

site	Reco	Ra[(a)]	Rsoil[(b)]	Rleaf[(c)]	Rwood[(d)]	Rroot[(e)]			Ru[(f)]
						coarse	fine	total	
Caxiuanã[4]	3205	1851	1612	502	871	183	295	478	NA
Changbai Mountains[6]	1242	NA	593	264	385	NAs	NAs	NAs	NAs
Chibougamau EOBS[9]	1032	492	710	128	126	NAs	NAs	170	68
Collelongo[11]	764	NA	428	275	61	NAs	NAs	NAs	0
Duke Forest[13]	1908	1703	928	492	488	61	662	723	NAs
Hesse[21]	1199	801	663	194	282	NAs	NAs	325	0
Huhus[25]	793	NA	497	224	72	NAs	NAs	NAs	NA
Jacaranda[27]	3210	NA	1210	980	420	NAs	NAs	NAs	150
Marys River Fir[28]	2009	NA	1205	422	382	NAs	NAs	NAs	NA
Metolius[30,31]	894	673	683	157	54	NAs	NAs	462	NAs
Prince Albert SSA SOAS[38,39]	1492	901	905	464	123	214	100	314	NAs
SMEARII[41]	919	NA	607	252	61	NAs	NAs	NAs	NAs
Sylvania hardwood[19,44]	1013	743	724	115	131	NAs	NAs	497	0
Tapajos km 67[5]	2770	1490	1200	740	380	NAs	NAs	370	NA
Tumbarumba[51]	1452	845	876	405	170	34	236	270	NAs
TurkeyPointTP02[53,54]	773	504	539	234	1	NAs	NAs	269	NA
TurkeyPointTP89[53,54]	1985	1713	511	1203	271	NAs	NAs	239	0
TurkeyPointTP74[53,54]	1278	948	558	527	193	NAs	NAs	228	NA
TurkeyPointTP39[53,54]	1526	1128	671	726	129	NAs	NAs	273	NA
University of Michigan[55,56]	1449	931	1036	246	167	NAs	NAs	518	NAs
Walker Branch[57,58]	1625	1097	882	409	247	NAs	NAs	441	NAs
Willow Creek[59]	1251	784	890	57	225	NA	502	502	NA
Wytham Woods[63]	2027	1493	619	716	689	NA	88	88	NA

Xishuangbanna[65]	2242	1720	885	955	334	NAs	NAs	431	NAs
Yatir[67,68]	731	NA	440	228	63	NAs	NAs	NAs	NA

(a) ecosystem autotrophic respiration; (b) total soil respiration; (c) leaf respiration; (d) aboveground wood respiration; (e) root respiration; (f) understory respiration. Green cells: data available; NAs: data not available separately but aggregated in other components (e.g. Reco or total Rroot); yellow cells and NA: data not available.

Supplementary Table 4. Location and characteristics of the study sites.

site and references	location			climate			canopy			fertility[g]	elevation variability[h]	slope[i]
	country	latitude	longitude	zone[a]	MAT[b]	MAP[c]	leaf type[d]	leaf habit[e]	LAI[f]			
Caxiuanã[5,69]	BR	-1.7197	-51.4590	Tr	26.9	2314	BRO	EVE	5.3	I	11.0	2.5
Changbai Mountains[6]	CN	42.4025	128.0958	Te	3.6	700	MIX	MIX	6.2	F	16.8	2.7
Chibougamau EOBS[8,9]	CA	49.693	-74.432	B	0	961	NED	EVE	3.7	I	5.9	2.5
Collelongo[11,19]	IT	41.8494	13.5881	Te	7.1	1104	BRO	DEC	5.2	F	109.9	25.3
Dooray[12]	IE	52.95	-7.25	Te	9.3	850	NED	EVE	8.7	F	29.7	7.4
Duke Forest[29,70]	US	35.9782	-79.0942	Te	15.6	1064	NED	EVE	5.2	I	10.6	4.3
Fujiyoshida[17,71]	JP	35.4514	138.7653	Te	9.7	2025	NED	EVE	5.5	I	44.1	5.5
Hainich[18]	DE	51.0793	10.4520	Te	7.75	775	BRO	DEC	4.8	F	31.5	5.6
Harvard[20]	US	42.5378	-72.1715	Te	7.1	1066	BRO	DEC	4	I	21.0	5.5
Hesse[23]	FR	48.6742	7.0656	Te	9.2	820	BRO	DEC	5.6	F	17.3	3.3
Huhus[19,25,72]	FI	62.87	30.82	B	2.0	724	NED	EVE	2.1	I	NA	NA
Lageren[26]	CH	47.478	8.36533	Te	7.4	1000	MIX	MIX	5.5	M	99.2	24.0
Jacaranda[5,69]	BR	-2.6091	-60.2093	Tr	27.1	2272	BRO	EVE	5.3	I	19.1	3.3
Marys River Fir[28,73]	US	44.6465	-123.5515	Te	9.8	1557	NED	EVE	9.4	F	42.2	9.5
Metolius [30,31]	US	44.42	-121.67	Te	8.4	370	NED	EVE	1.5	M	96.5	23.7
Morgan Monroe[20]	US	39.3232	-86.4131	Te	11.1	1012	BRO	DEC	4.9	M	16.5	4.7
NAU Centennial Thinned[33]	US	35.1426	-111.7273	Te	9.3	632	NED	EVE	1.2	M	18.2	6.2
NAU Centennial Undisturbed [33]	US	35.0890	-111.7620	Te	9	684	NED	EVE	2.2	M	22.1	4.0
Prince Albert SSA SOJP[34,74]	CA	53.916	-104.69	B	0.5	406	NED	EVE	1.3	I	4.9	2.1
Prince Albert SSA SOAS[74]	CA	53.629	-106.2	B	0.5	406	BRO	DEC	2.4	I	8.1	2.1
SMEARII[41]	FI	61.85	24.28	B	4.3	648	NED	EVE	6.3	M	NA	NA
Sylvania hardwood[42]	US	46.242	-89.3477	B	3.9	771	MIX	MIX	4.1	I	9.0	2.8
Takayama[45,71]	JP	36.1462	137.4231	Te	7.3	2400	BRO	DEC	3.5	F	92.0	19.7
Tapajos km 67[5,75]	BR	-2.8567	-54.9589	Tr	25.9	2091	BRO	EVE	5.3	I	2.7	3.8
Thompson NSA NOBS[49,76]	CA	55.88	-98.48	B	0.8	439	NED	EVE	4.4	I	6.1	1.3
Tumbarumba[51,77]	AU	-35.6557	148.1521	Te	9.2	1011	BRO	EVE	1.38	M	27.6	6.4
Turkey Point TP02[53,54]	CA	42.6609	-80.5595	Te	7.8	1010	NED	EVE	1	F	7.0	2.0
Turkey Point TP89[53]	CA	42.7744	-80.4588	Te	7.8	1010	NED	EVE	12.8	F	7.1	1.8
Turkey Point TP74[53]	CA	42.7068	-80.3483	Te	7.8	1010	NED	EVE	5.9	M	6.0	1.0
Turkey Point TP39[53]	CA	42.7098	-80.3574	Te	7.8	1010	NED	EVE	8	M	6.1	1.5
University of Michigan[55]	US	45.5598	-84.7138	Te	5.5	817	BRO	DEC	3.5	I	12.9	2.8
Walker Range[20]	US	35.9588	-84.2874	Te	13.8	1352	BRO	DEC	6.2	I	23.6	3.9
Willow Creek[20]	US	45.8058	-90.0797	B	4.8	776	BRO	DEC	4.2	F	7.3	2.1
Wind River[61,78]	US	45.8205	-121.9519	Te	8.7	2467	NED	EVE	6.92	M	26.2	13.8
Wytham Woods[63,64]	UK	51.46	-1.32	Te	10.1	730	BRO	DEC	7.8	F	37.4	7.9
Xishuangbanna[65,79]	CN	21.9275	101.2653	Tr	21.7	1487	BRO	DEC	5.5	I	20.2	8.3
Yamashiro[66,80]	JP	34.7948	135.8462	Te	15.5	1449	BRO	MIX	3	I	62.5	11.4
Yatir[68,81]	IL	31.347	35.052	Te	17.6	275	NED	EVE	1.6	I	25.1	4.2

(a) climatic zone: Bo: boreal, Te: temperate and Tr: tropical; (b) mean annual temperature (°C); (c) mean annual precipitation (mm y^{-1}); (d) needleleaved (NED), broadleaved (BRO) or mixed (MIX); (e) evergreen (EVE), deciduous (DEC) or mixed (MIX); (f) leaf area index (m^2 leaf m^{-2} ground); (g) fertile (F), moderately fertile (M) and infertile (I); (h) elevation variability refers to the standard deviation of the elevation (m) of 729 pixels composing a 2430 × 2430 m quadrat centered around the EC tower, and (i) the slope (%) was derived from the elevation and distance of the highest and lowest pixels within the quadrat in (h). NA: data not available.

Supplementary Table 5. Net ecosystem production (gC m^{-2} y^{-1}) from biometric methods based on temporal differences in ecosystem carbon stocks ($NEP_{BM-\Delta S}$) and eddy-covariance (NEP_{EC}).

	measurement period	$NEP_{BM-\Delta S}$								NEP_{EC}
		Total	Phytomass				Necromass		Soil	
			wood[(a)]	leaves	fine roots	understory	litter	cwd[(b)]		
Dooray[12]	2002	1346	1051	NA	43	NA	NA	137	115[(c)]	890
Brasschaat[82]	2002-2010	175	206	3	0	34	-72	2	1	250
Harvard[83]	1993-2000	160	100	NA	NA	NA	NA	40[(d)]	20[(e)]	200
HBS00[84]	2002-2008	-92	0	NA	NA	NA	-18	-41	-34	-118
Morgan Monroe[20]	≤1999	320	320	NA	NA	NA	NA	NA	0	236
Walker Branch[20]	1972-1999	264	264	NA	NA	NA	NA	NA	0	577
Lageren[26]	2006-2009	429	407	NA	NA	NA	NA	NA	22	435
Yamashiro[66]	1999-2003	172	130	NA	NA	NA	NA	11	31	123

(a) including stem, branches and coarse roots; (b) coarse woody debris; (c) soil stock difference derived from current soil stocks of the plantation and soil stocks of a grassland similar to the site before planting; (d) derived from input (mortality, turnover) and output (decomposition); (e) derived from the residence time of ^{14}C at the site. NA: data not available.

Supplementary Table 6. Impact of the spatial scaling factor for wood respiration rate (wood area or wood volume) and of the leaf type (needleleaved, broadleaved or mixed) on the annual estimate of wood respiration and leaf respiration (Rwood and Rleaf, respectively) and the proportion of Rwood and Rleaf to the total ecosystem respiration, Reco (Rwood:Reco and Rleaf:Reco, respectively).

Upscaling factor	Rwood		Rwood:Reco		Rleaf		Rleaf:Reco	
	p	R^2	p	R^2	p	R^2	p	R^2
Wood scaling factor	0.34	0.073	0.77	<0.01	n.a.	n.a.	n.a.	n.a.
Leaf type	n.a.	n.a.	n.a.	n.a.	0.20	0.78	0.29	0.12

n.a.: not applicable

Supplementary Table 7. Components of net primary production (NPP), autotrophic respiration (Ra), heterotopic respiration (Rh) and soil respiration (Rsoil) considered for the study sites and their classification as necessary (neces), ancillary (ancil) or no needed (no) for the site inclusion in the dataset of net ecosystem production (NEP), ecosystem respiration (Reco) and gross primary production (GPP).

	NEP			Reco			GPP		
	neces	ancil	no	neces	ancil	no	neces	ancil	no
NPP									
Foliage	x					x	x		
Above wood	x					x	x		
Fine roots	x					x	x		
Coarse roots	x					x	x		
Understory		x				x		x	
Branch turnover		x				x		x	
Reproductive organs		x				x		x	
Herbivory		x				x		x	
Mycorrhizae		x				x		x	
Other[(a)]		x				x		x	
Ra									
Foliage (Rleaf)			x	x			x		
Above wood (Rwood)			x	x			x		
Roots (Rroot)			x	x			x		
Understory (Ru)			x		x			x	
Rh									
Soil heterotrophic (Rh-soil)	x					x			x
Coarse woody debris (Rh-cwd)		x			x				x
Rsoil			x	x					x

(a) production of volatile organic compounds, epiphytes and dissolved organic carbon

Supplementary Table 8. Characteristics of the allometric relationships (AR) used to measure the standing biomass of above- (stem and branches) and belowground wood (coarse roots) and of the method used to measure leaf production (litter traps, LT, or AR) at the sites with biometric measurements of net primary production.

Site	AR aboveground wood			AR belowground wood			AR quality[(a)]	leaf production
	species-specific	site-specific	variable[(b)]	species-specific	site-specific	variable[(b)]		
Caxiuanã[4]	no	no	D, TH, WD	no	no	fixed[(c)]	L	LT
Chibougamau EOBS[9]	yes	no	D, TH	yes	no	D	M	AR[(d)]
Dooray[12]	yes	no	D, TH[(e)]	yes	no	D, TH[(e)]	M	LT
Duke Forest[13]	yes	yes	D	yes	yes	D	H	LT
Fujiyoshida[17]	yes	yes	D	yes	yes	D	H	LT
Hainich[18]	NA	NA	NA	NA	NA	NA	NA	LT
Harvard[20]	yes	no	D	yes	no	D	M	LT
Hesse[23]	yes	yes	D	yes	yes	D	H	LT
Lageren[26]	yes	no	D, TH, WD, E	yes	no	D, WD, A	M	LT
Metolius[30,31]	yes	no	D	no	no	[(f)]	L	AR[(g)]
Morgan Monroe[32]	yes	no	D	yes	no	D	M	LT
NAU Centennial Thinned[33]	yes	yes	D	yes	no	D	M	LT
NAU Centennial Undisturbed[33]	yes	yes	D	yes	no	D	M	LT
Prince Albert SSA SOJP[34]	yes	yes	D	yes	yes	D	H	AR[(h)]
Prince Albert SSA SOAS[37,40]	yes	yes	D	yes	no	D	M	LT
Sylvania hardwood[43,44]	NA	NA	NA	NA	NA	NA	NA	LT
Takayama[45]	yes	yes	D	yes	yes	D	H	LT
Tapajos km 6[5,85]	no	no	D, WD	no	no	fixed[(i)]	L	LT
Thompson NSA NOBS[49]	yes	yes	D	yes	yes	D	H	LT
Tumbarumba[51]	yes	yes	D	yes	yes	D	H	LT
TurkeyPointTP02[54]	yes	yes	D	yes	yes	D	H	LT
TurkeyPointTP89[54]	yes	yes	D	yes	yes	D	H	LT
TurkeyPointTP74[54]	yes	yes	D	yes	yes	D	H	LT
TurkeyPointTP39[54]	yes	yes	D	yes	yes	D	H	LT
University of Michigan[55]	yes	no	D	no	no	fixed[(j)]	L	LT
Walker Range[57]	yes	yes	D	no	no	fixed[(c)]	M	LT

Willow Creek[20]	yes	no	D	no	no	fixed[(c)]	L	LT
Wind River[62]	yes	no	D	yes	no	D	M	LT
Wytham Woods[63]	yes	no	D	no	no	fixed[(c)]	L	LT
Xishuangbanna[65]	no	yes	D	no	yes	D	L	LT
Yamashiro[66]	yes	yes	D	no	yes	D	M	LT

(a) H: high, M: moderate, L: low (see Methods for details); (b): tree and stand characteristics representing the independent / driving variable(s) in the allometric relationships, with D: tree diameter, TH: tree height, WD: wood density, E: site elevation, and A: stand age; (c): fixed fraction of aboveground wood production (20-21%); (d): leaf production-to-stem production ratio; (e): equation for whole tree (aboveground + belowground); (f): simulations from process-based model; (g): proportion of current year vs. old leaves biomass; (h): relationship with diameter; (i): same production-to-biomass ratio as for aboveground wood, and (j): relationship above vs. belowground wood.

Supplementary Table 9. Amplitude of confidence interval (equivalent to four times the s.e.m) approximated following Luyssaert et al. (2007)[19] (approx) or directly estimated (estim) for the net ecosystem production (NEP, gC m^{-2} y^{-1}), ecosystem respiration (Reco, gC m^{-2} y^{-1}) and gross primary production (GPP, gC m^{-2} y^{-1}) from biometric methods on forests of the boreal (B), temperate (Te) or tropical (Tr) zone.

site	Climate	NEP		Reco		GPP	
		approx	estim	approx	estim	approx	estim
Chibougamau EOBS[9]	B	NA	NA	774	714	774	428
Caxiuanã[4]	Tr	NA	NA	590	1646	590	1356
Dooray[12]	Te	210	414	NA	NA	NA	NA
Jacaranda[5]	Tr	NA	NA	588	1842	NA	NA
Tapajos67[5]	Tr	NA	NA	586	1724	586	1724
Tumbarumba[51]	Te	NA	NA	446	290	446	290
Wind River[62]	Te	420	534	NA	NA	NA	NA
Wytham Woods[63]	Te	NA	NA	756	588	756	628

NA: not available

Supplementary Table 10. Contribution of mycorrhiza production to total stand net primary production (NPP) from field- and culture studies. Data are reported for each site (for field studies), with mean ± s.e.m, minumum and maximum values, and number of replicates (for both field- and culture studies).

Forest type and reference	% NPP
Abies amabilis (23 y old)[86]	14%
Abies amabilis (180 y old)[86]	15%
Pinus sylvestris[87,88]	15%
Mixed conifer-deciduous[89]	21%
Piñon-juniper[90]	18%
Pinus taeda[15]	0.3%
Average field-studies (this table)	14±3% (0.3-21%, n=6)
Average culture-studies[88]	9±1% (1-21%, n=33)

Supplementary Methods

We report here a summary of the methodological approach used at each site for the biometric determination of the production and respiratory components to estimate NEP, Reco and GPP. Thus, the document provides site overviews about measurement techniques, protocols, measurement periods, replicates, data processing etc. However, note that this text does not necessary include all methodological information extracted from the literature for a given site (see Supplementary Data 1 for the dataset used in the analysis) and, according to the information available, might contain information on a given process or variable only for a portion of the sites.

Caxiuanã

Net primary production (from Doughty et al 2014[4]; otherwise indicated)
Stem and branches: allometry
Coarse roots: assumed as 21% of aboveground wood production
Leaves: litter traps (collected each 14 days – litter decomposition in canopy not taken into account)
Fine roots: in-growth cores
Understory: not measured for trees with diameter < 2.5 cm
Reproductive material: litter traps (collected each 14 days)
Branch turnover: taken into account (survey each 2 months)
Herbivory: image analysis of leaf damage by herbivores (each month)
VOC: empirical – combination from different sites[5]
DOC: empirical – combination from different sites[5]

Respiration (from Doughty et al 2014[4]; otherwise indicated)

Methods
Soil: closed dynamic chambers + infra-red gas analyzer (IRGA) (July 2009 – April 2011, monthly); replicates: n=25
Fine roots: trenching experiment (3 plots: control, no roots no mycorrhiza, no roots yes mycorrhiza)
Soil – heterotrophic component: Rsoil-Rroot
Stem: closed dynamic soil chambers + IRGA (July 2009–December 2010, every 2 months); replicates: n=25 trees, 1.3 m height.
Coarse roots: assumed 21% of aboveground wood respiration
Leaves: NIGHTTIME: leaf dark respiration on cut branches with cuvette + IRGA (measurements during 2 months Jan-Feb 2007); replicates: n=30 trees, for each tree, one branch sunlit and one shaded. DAYTIME DATA: daytime respiration was assumed to be only 33% of the nighttime respiration to account for daytime photoinhibition of leaf dark respiration[5]
Understory: not accounted
Coarse woody debris: derived from data on amount of wood falling due to mortality multiplied by 76% (amount of dead wood respired away before entering the soil; this factor, 76%, is from another site)[5]

Scaling
Soil:
TEMPORAL: averaged per month with no consideration of seasonality
SPATIAL: basic calculation
Stem:
TEMPORAL: bimonthly averages with no consideration of seasonality (little seasonal variation in stem respiration was documented[5]
SPATIAL: stem area (considering BRANCHES and that stem respiration constant with height). Relationship between woody NPP and woody respiration: the trees measured for woody respiration grew faster than the average trees in the plot. Therefore, when scaled to the plot level, respiratory fluxes were reduced by 11%.
Leaves:

TEMPORAL: The wet season respiration mean was applied to all months with >100 mm rain; for the dry season, measured dry season respiration was linearly scaled by the soil moisture saturation
SPATIAL: LAI.

Changbai Mountains

Net primary production
No data available meeting quality standard for our analysis

Respiration[6]

Methods
Soil: chambers with offline CO_2 measurements (gas chromatography) on surface with cut understory (9-11 a.m., each 4–8-day from April to November, monthly from December till March, in 2003); replicates: n=6
Stem: chambers with online CO_2 measurements (LI-6400) (3-7-day intervals from May to December, in 2003); replicates: n=6, for 4 main tree species.
Leaves: CO_2 exchange with LI-6400 at night (9:00–11:00 p.m., monthly from May to September, in 2003)
Understory: chambers with offline CO_2 measurements (gas chromatography) at night
Coarse woody debris: not measured.

Scaling:
Soil:
TEMPORAL: relationship with temperature (seasonal and site data)
SPATIAL: basic calculation
Stem:
TEMPORAL: relationship with temperature (seasonal and site data)
SPATIAL: wood volume (comprising BRANCHES)
Leaves:
TEMPORAL: relationship with temperature (seasonal and site data)
SPATIAL: LAI / leaf biomass
Understory:
TEMPORAL: relationship with temperature (seasonal and site data)
SPATIAL: LAI / leaf biomass

Chibougamau EOBS

Net primary production[9]
Stem: allometry, average of 2003-2008 (2005 as measurement year)
Coarse roots: allometry, average of 2003-2008 (2005 as measurement year), assuming absence of coarse root mortality and turnover (as in all sites)
Branches: allometry
Leaves: allometry
Fine roots: product of standing biomass at the site (soil cores in 2005, 2006 and 2007) and fine root turnover rate from a nearby site (minirhizotron and fine root biomass from root cores)
Understory: nonvascular: 50% of GPP; vascular: present (e.g. shrubs) but not accounted for.

Respiration[9]

Methods
Soil (comprising nonvascular): automated chamber system (May to October 2005; n=6-9 but different nonvascular understory)
Fine root respiration: Rsoil - Rh-soil
Soil – heterotrophic component: trenching method (data from 2005)
Stem: chambers+ LI-COR, both automatic (4 periods growing season 2005) and manual (7 times during growing season 2005); replicates: breast height: n=8-12 trees for 2 species, and crown: n=2-6 trees and 2 species.
Branches: chambers + LI-COR, both automatic (4 periods growing season 2005) and manual on cut branches (7 times during growing season 2005); replicates: n=1-6 trees for 1-2 species (branches analyzed: 2 automatic and 82 manual)
Leaves: LCA-4 portable gas exchange system on 1-year-old shoots on cut branches (4 days in August and September 2005); replicates: n=3-4 trees and 2 branches for 2 species; 21 shoots per species analyzed; estimate growth respiration added (coefficient × biomass new leaves)
Understory: nonvascular: in Rsoil; vascular: not measured.
Coarse woody debris: not mentioned / not measured

Scaling:
Soil:
TEMPORAL: classical relationship with temperature and Q_{10} (seasonal and site data)
SPATIAL: type ground cover and site ground cover proportion taken into account; correction of 16% because previous research detected this error
Stem:
TEMPORAL: classical relationship with temperature and Q_{10} (seasonal and site data)
SPATIAL: stem surface
Branches:
TEMPORAL: classical relationship with temperature but Q_{10} assumed (not measured)
SPATIAL: dry mass (branch biomass in six branch diameter classes from inventory/allometry)
Leaves:
TEMPORAL: classical relationship with temperature but Q_{10} assumed (not measured)
SPATIAL: dry mass

Collelongo

Net primary production
Not available for study period considered (2007)

Respiration[11]

Methods
Soil: Chambers with closed dynamic system (EGM 4, PP-System, Hitchin, UK) (May 2007 until May 2008, each 2-6 weeks for 1 year except between December to March when snow covered the soil); replicates: n=50, 5 collars in 10 plots
Stem: Cuvette + LCA-4 open-system infrared gas analyzer (Analytical Development Company, Hoddeson, UK) (growing season of 2007 + winter campaign in February 2008); replicates: n=10 trees
Leaves: detached leaves + portable gas exchange system (LiCor 6400) (measurements on one occasion in July 2007, during three periods in the day); replicates: 10 shade and 10 sun leaves (fully expanded leaves)
Understory: negligible
Coarse woody debris: negligible

Scaling
Soil:
TEMPORAL: relationship with temperature and soil water content
SPATIAL: basic calculation
Stem:
TEMPORAL: relationship with temperature
SPATIAL: woody surface area (considering also BRANCHES)
Leaves:
TEMPORAL: temperature response based on data from literature[91]
SPATIAL: leaf area index (LAI); the partition of LAI into sun and shaded leaves based on a precedent study carried out at the site

Dooray

Net primary production[12]
Stem and branches: allometry, 2002-2003
Coarse roots: allometry, 2002-2003
Leaves: litter traps + allometry, 2002-2003
Fine roots: ingrowth cores, 2003
Understory: not mentioned
Branch turnover: considered

Respiration[12]

Methods
Soil: automatic chamber + IRGA, used to check the model for seasonal Rsoil (120 days but no info on the year)
Soil – heterotrophic component: trenching experiment: soil respiration measurements on 30 cm deep collars that killed all roots (validated models for 2002-2003)
Coarse woody debris: negligible but aboveground dead considered in this category: chamber + IRGA of dead branches and needles in laboratory

Scaling
Soil:
TEMPORAL: function of soil temperature and soil moisture content
SPATIAL: basic calculation
Coarse woody debris:
TEMPORAL: temperature function
SPATIAL: basic calculation from surveys of standing aboveground dead

Duke Forest

Net primary production (from Hamilton et al 2002[13]; otherwise indicated)
Stem and branches: allometry
Coarse roots: allometry
Leaves: allometry + litter traps
Fine roots: bi-weekly soil cores, including estimates of root mortality and decomposition
Understory: included with overstory
Reproductive material: litter traps
Herbivory: accounted for
Dissolved inorganic/organic carbon: accounted for
Mycorrhizal transfer: accounted for[15]

Respiration (from Hamilton et al 2002[13]; otherwise indicated)

Methods
Soil: Soda lime (monthly from January 1997 onwards, daily measurements); replicates: n=4 for 3 plots[92]
Fine roots: MAINTENANCE: measurements on roots still attached to the tree using a portable gas exchange system in July 2000 (other year than measurement year 1998); GROWTH: calculated as 25% of total yearly fine root production
Soil – heterotrophic component: Rsoil – Rroot
Stem: MAINTENANCE: cuvette + automated open-system infrared gas-analysis systems (in October at end of growth); replicates: n=5 trees in 3 plots; GROWTH: from measurements of heat of combustion and the fraction of carbon, nitrogen and ash in each sample.
Branches: MAINTENANCE: as for stem, but respiration rates for branch sapwood were assumed to be 2.52 times higher than for stem; GROWTH: as for stem
Coarse roots: as for stem, for both MAINTENANCE and GROWTH
Leaves: MAINTENANCE: night respiration of detached leaves using an open gas-exchange system with a conifer cuvette (LI 6400) (mid-June, late July, early September 1999, other year than measurements year 1998, 21.00 - 04.00 h); replicates: n=3 samples from 3 trees, for 2 species; taken into account higher respiration in top canopy than lower canopy and that 75% sun and 25% shade leaves (for pine – dominant) and 100% shade (for sub-dominated); PHOTOINHIBITION: assumed that the respiration rate during the day was 60% of the rate during the night; GROWTH: from measurements of heat of combustion and the fraction of carbon, nitrogen and ash in each sample[93].
Understory: included in overstory
Coarse wood debris: negligible

Scaling
Soil:
TEMPORAL: temperature response function
SPATIAL: basic calculation
Stem:
TEMPORAL: temperature response function: Q_{10} from measurements; base respiration rate: from literature (generic, no species-specific)
SPATIAL: sapwood volume (also for BRANCHES)
Leaves:

TEMPORAL: temperature response function : Q_{10} and base respiration rate from measurements;
SPATIAL: proportion sun/shade leaves
Fine roots:
TEMPORAL: temperature response function; Q_{10} from literature and base respiration rate measured
SPATIAL: biomass
Coarse roots:
TEMPORAL: temperature response function: Q_{10} and base respiration rate like stem
SPATIAL: sapwood volume

Fujiyoshida

Net primary production[17]
Stem and branches: allometry, 1999-2008
Coarse roots: allometry, 1999-2008
Leaves: allometry + litter traps, 1999-2007
Fine roots: sequential monthly core sampling, 2000
Understory: not measured for trees <5 cm DBH
Reproductive material: accounted for (litter traps), 1999-2007
Branch turnover: accounted for through surveys, 2000-2004
Twigs (and bark): accounted for (litter traps), 1999-2007

Respiration[17]

Methods
Soil: chambers + LiCor 6200 gas exchange analyzer (monthly intervals in 2006-2007, measurements at noon); replicates: n=20
Fine roots: fresh cut roots in chambers (December 2006, May, September, October and December 2007) on fine <2 mm and middle size roots >2 mm
Soil – heterotrophic component: Rsoil - Rroot
Coarse woody debris: from amount dead branches from surveys, assuming that dead woody debris pool constant

Scaling
Soil:
TEMPORAL: temperature function
SPATIAL: basic calculation taking into account presence lava flow (17.6% area)
Fine roots:
TEMPORAL: temperature function
SPATIAL: biomass of fine and middle size roots, separately, taking into account presence of lava flow (17.6% area)

Hainich

Net primary production
Stem and branches: allometry, 2000-2002[19]
Coarse roots: allometry, 2000-2002[19]
Leaves: litter traps, 2002[18]
Fine roots: sequential coring, 2002[18,94]
Understory: sequential harvesting, 2002[18,94]
Reproductive material: accounted for, 2002[18]

Respiration

Methods
Soil: see below Rh-soil
Soil – heterotrophic component: component integration method for 2002, with laboratory incubation and upscaling using field data[18]
Coarse woody debris: not measured

Scaling
Soil heterotrophic respiration:
TEMPORAL: temperature and moisture response function[18]
SPATIAL: respiration expressed as g CO_2-C g^{-1} C soil; upscaled using amount of C per soil layer in 1 m^2 soil[18]

Harvard Forest

Net primary production
Stem and branches: allometry, 1999[20]
Coarse roots: allometry,1999[20]
Leaves: litter traps, 1999[20]
Fine roots: sequential coring, 1979[95]
Understory: allometric equations for woody vegetation and biomass harvest for herbaceous vegetation (no time info)[20]
Reproductive material: litter traps, 1999[20]

Respiration

Methods
Soil: chambers + infrared gas analyzer (June 1995 - May 1996, 9.00-13.00 am, each 1-3 weeks interval except when snow cover); replicates: n=36[96]
Soil – heterotrophic component: assumed as 50% of Rsoil[97]
Coarse woody debris: not measured

Scaling
Soil:
TEMPORAL: temperature and moisture function[96]
SPATIAL: basic calculation, with taking into account different vegetation cover areas [20,96]

Hesse

Net primary production
Stem and branches: allometry, 1997[21]
Coarse roots: allometry, 1997[21]
Leaves: litter traps, 1997[21]
Fine roots: ingrowth cores, 1997[22,24]
Understory: negligible

Respiration

Methods
Soil: chambers + LI-COR (each 2-4 weeks during June 1996 – November 1997); 6 plots with 12 measurements per plot during 8am – 4pm (24h measurement campaign confirmed 8h was ok)[98]
Fine roots: Rsoil - Rh-soil
Soil – heterotrophic component: trenching + modelling[99]
Stem and branches: chambers + IRGA (STEM: monthly for whole year 1997, n=15 trees; BRANCHES: May to October 1997, continuous; 3 branches within 1 tree), both maintenance and growth respiration accounted for[100]
Leaves: branch bag + IRGA (2 bags, one top and one down in canopy May to October 1997, continuous); both maintenance and growth respiration are accounted for; photo-inhibition accounted for[21]
Understory: negligible
Coarse woody debris: data provided without details on methodology[23]

Scaling:
Soil:
TEMPORAL: relationship based on soil water content and temperature[98]
SPATIAL: basic calculation
Stem/branches:
TEMPORAL: classical relationship with temperature and Q_{10} (seasonal and site data)[100]
SPATIAL: wood volume
Leaves
TEMPORAL: classical relationship with temperature and Q_{10} (seasonal and site data)[21]
SPATIAL: LAI/leaf biomass

Huhus

Net primary production
Not available

Respiration[25]

Methods
Soil: automated chambers (April to November each year from 2001 to 2004); replicates: n = 4
Stem: automated chambers (April to November each year from 2001 to 2004); replicates: n=3 trees (as most of studies, assumed that efflux constant along the stem)
Leaves: automated chambers (April to November each year from 2001 to 2004); replicates: n=4 branches different position of 4 trees
Understory: negligible
Coarse woody debris: not measured.

Scaling
Soil:
TEMPORAL: temperature functions (seasonal and site based)
SPATIAL: basic calculation
Stem:
TEMPORAL: temperature functions (seasonal and site based)
SPATIAL: stem volume (BRANCHES not included)
Leaves:
TEMPORAL: temperature functions (seasonal and site based)
SPATIAL: leaf area index

Jacaranda

Net primary production
No data available meeting quality standard for our analysis

Respiration (Chamber et al 2004[27]; otherwise indicated)

Methods
Soil: chamber + IRGA system (8 times during the day (08.00–16.00 h) between July 2000 and June 2001 at 4-6-week intervals); replicates: n=54
Stem: chambers + infrared gas analyzer (IRGA) (8 times between August 2000 and June 2001 at 4-6 week intervals); replicates: 50 trees from five growth classes
Leaves: dark chambers + LiCor 6400 (from April to November 2001, hourly intervals over a combined 24-h period for the same leaf); leaves from 20 large trees (>14 m in height). Daytime flux was reduced by 40% to account for photoinhibition.
Understory: taken from another tropical site (Pasoh, Malesia) from the literature
Coarse woody debris: chambers + IRGA and surveys (1996-1997)[101]; estimates for smaller debris (fine woody debris) available at the site

Scaling
Soil:
TEMPORAL: likely average per month (no seasonality)
SPATIAL: basic calculation
Stem:
TEMPORAL: likely average per month (no relationship between Rwood and stem surface temperature)
SPATIAL: wood area (including BRANCHES) and taking into account growth rate per tree (5 classes) and number of trees within each 5 classes
Leaves:
TEMPORAL: likely average per month (no seasonality)
SPATIAL: leaf area index

Lageren

Net primary production [26]
Stem and branches: allometry, 2006-2009
Coarse roots: allometry, 2006-2009
Leaves: allometry + litter traps, 2006-2009
Fine roots: standing root biomass (estimated as 50% of foliage biomass based on literature) + turnover measured on site from maximum fine root biomass (sequential coring) and annual fine root growth (ingrowth cores)
Understory: accounted for from litter production
Reproductive material: allometry, 2006-2009
Twigs: accounted for (allometric 2006-2009)

Respiration[26]

Methods
Soil: chambers + IRGA (2006 to 2009, every 2–3 weeks), automatic : n=1, and manual: n=17
Soil – heterotrophic component: two methods: root exclusion and root excised (from September 2006 till September 2007, root exclusion also likely from March 2006 till May 2008)[102]
Coarse woody debris: not measured

Scaling
Soil:
TEMPORAL: function of temperature[102]
SPATIAL: basic calculation
Soil – heterotrophic component:
TEMPORAL: function of temperature[102]
SPATIAL: basic calculation

Marys River Fir

Net primary production
Not available

Respiration[28]

Methods
Soil: automatic chambers + infrared gas analyzer (Li-Cor 6262) (measured every 4 h continuously whole year but with gaps ca. 35% of time); replicates: n=6
Stem: chambers + ADC LCA3 open system (different 3-days periods along year to capture seasonality); replicates: n=3-8 trees, north side of trees
Leaves: portable photosynthesis system (Model LiCor 6400) on branches cut during night and measured in lab between 06:00 and 09:00 am (measured on one occasion end August 2011); replicates: n=8 branches from 4 trees, mid to upper canopy. Correction factor 1.13 applied to base respiration to fit global relationship between leaf N and respiration rate.
Understory: sparse, not measured
Coarse woody debris: not measured.

Scaling
Soil:
TEMPORAL: gaps were interpolated (when <4 h) or filled with a temperature and soil moisture model (when > 8h) validated at the site
SPATIAL: upscaling done using extensive data from 3 to 5 periodic spatial surveys per year on 20 separate locations
Stem:
TEMPORAL: temperature function
SPATIAL: sapwood volume (including BRANCHES)
Leaves:
TEMPORAL: temperature function, with base respiration rate (R_{20}) made as function of N (varying seasonally) and with Q_{10} dependent on temperature (4-day running mean temperature)[91]
SPATIAL: leaf mass and PAI (plant area index)

Metolius

Net primary production
Stem and branches: allometry, 1996[30]
Fine + coarse roots: derived from measurements of aboveground NPP and simulation of aboveground vs belowground NPP (average of 3-PG and PnNET-II_{300} simulations)[31]
Leaves: from specific leaf area (m2 leaf g-1 dry weight) and mean leaf area of newly expanded foliage, which was determined from a subsample of branches from at least 12 trees (in 1996)[30]
Understory: NPP determination not mentioned

Respiration

Methods
Soil: chambers + LICOR 6200 infrared gas analyzer (IRGA) (23 measurements during the year, March 1996-March 1997)[30]
Fine roots + coarse roots: derived from the total belowground flux (which is root NPP + Rroot, [30,103]) with subtraction of root NPP (see above)
Soil – heterotrophic component: Rsoil - Rroot
Stem: Chambers + ADC LCA3 open system (on five days from January to October 1996; days of year 9, 156, 184, 213, and 284); replicates: n=10 young trees and n=10 old trees, on north side of tree[30]
Leaves: LI-COR 6400 open system + ADC LCA3 open system; nocturnal respiration measured four days through the year 1996 (in February, April, June, July); replicates: n=4-6 of 2 trees, for needles 1 year-old (in July, 3 age needle classes at 3 heights in the canopy were measured: based on these results they did not estimate respiration of expanding foliage separately and assumed that age class 1 represented the mean for all classes)[30]
Understory (mainly strawberry, *Fragaria vesca)*: derived from *P. ponderosa* data corrected for seasonal changes in the fraction of daytime respiration (photosynthetic light response at 0 PAR) by *F. vesca* vs. *P. ponderosa* (for 1996)[30]
Coarse woody debris: not mentioned

Scaling[30]
Soil:
TEMPORAL: temperature response function
SPATIAL: basic calculation
Stem:
TEMPORAL: temperature response function
SPATIAL: sapwood volume (including BRANCHES)
Leaves:
TEMPORAL: temperature response function
SPATIAL: hemi-surface area (HSA; one-half the total surface area)
Understory: as for Leaves

Morgan Monroe

Net primary production [32]
Stem and branches: allometry, 1998-1999
Coarse roots: allometry, 1998-1999
Leaves: litter traps, 1998-1999
Fine roots: standing biomass + empirical model of Aber et al 1985[104]
Understory: harvesting in mid-summer of 1998-1999
Reproductive material: litter traps, 1998-1999
Herbivory: accounted, 1998-1999
Twigs: litter traps, 1998-1999

Respiration[32]

Methods
Soil: chambers + LiCor analyzer (each 2-3 weeks, 1998-1999, 10.00-14.00 h); replicates: n=50
Soil – heterotrophic component: assumed as 50% of Rsoil[97]
Coarse woody debris: surveys + decomposition rate from literature

Scaling
Soil:
TEMPORAL: function of temperature and water potential
SPATIAL: basic calculation

NAU Centennial Undisturbed

Net primary production [33]
Stem and branches: allometry, 2006-2007
Coarse roots: allometry, 2006-2007
Leaves: litter traps, 2006-2007
Fine roots: minirhizotron technique, 2006-2007
Understory: harvest peak biomass, 2006-2007

Respiration[33]

Methods
Soil: average of three methods: closed dynamic chambers, static chambers and soil CO_2 profiles (in 2007)
Soil – heterotrophic component: trenching experiment in 2005 in nearby similar site
Coarse woody debris: based on surveys and decomposition rate measured at the site

Scaling
Soil:
TEMPORAL: function of temperature and water content
SPATIAL: basic calculation

NAU Centennial Thinned

Net primary production [33]
Stem and branches: allometry, 2006-2007
Coarse roots: allometry, 2006-2007
Leaves: litter traps, 2006-2007
Fine roots: minirhizotron technique, 2006-2007
Understory: harvest peak biomass, 2006-2007

Respiration [33]

Methods
Soil: average of three methods: closed dynamic chambers, static chambers and soil CO_2 profiles (in 2007)
Soil – heterotrophic component: trenching experiment in 2005 in nearby similar site
Coarse woody debris: based on surveys and decomposition rate measured at the site

Scaling
Soil:
TEMPORAL: function of temperature and water content
SPATIAL: basic calculation

Prince Albert SSA SOAS

Net primary production
Stem and branches: allometry, 1994[37]
Coarse roots: allometry, 1995[40]
Leaves: litter traps, 1994[37]
Fine roots: minirhizotron technique, 1995[40]
Understory: harvesting, 1994[37]

Respiration

Method
Soil: closed gas exchange system including LI-6200 photosynthesis system and LI-6000-09 soil respiration chamber (late May-late September 1994, 10:00 -16:00 hour); replicates: n=20-30[38]
Fine roots: in situ: closed system + LI-COR 6200 (once during growing season); replicates: n=10-20 samples[39]
Soil – heterotrophic component: Rsoil - Rroot
Stem: automated chambers + LI-COR 6252 (several times between May – September 1994); replicates: n=16-20 trees; separation maintenance and growth respiration by subtracting the out-of-season respiration from the growing season respiration[39]
Coarse roots: assumed same rates as another, northern, site[39]
Leaves: chambers + closed system LI-6200 (June, July, August 1994); replicates: n=15-30 samples of 3-8 trees of 2 species[39]
Understory: LEAVES: chambers + closed system LI-6200 (5 sun and 5 shade leaves, 1994); WOOD: assumed as having same respiration rate as in northern site[39]
Coarse woody debris: not mentioned.

Scaling:
Soil[38]:
TEMPORAL: temperate response function
SPATIAL: different microsites taken into account
Stem[39]:
TEMPORAL: temperature response function at site
SPATIAL: sapwood volume
Leaves[39]:
TEMPORAL: temperature response function taken from same species in another, northern, site
SPATIAL: leaf area index
Understory[39]:
TEMPORAL: LEAVES and WOOD: temperature response function taken from same species in another, northern, site
SPATIAL: LEAVES leaf area index, and WOOD: sapwood volume
Roots[39]:
TEMPORAL: temperature response function taken from same species in another, northern, site
SPATIAL: biomass/volume

Prince Albert SSA SOJP

Net primary production [34]
Stem and branches: allometry, 1999-2000
Coarse roots: allometry, 1999-2000
Leaves: allometry for new leaves, 1999-2000
Fine roots: ingrowth core method, 1999-2000 (two years together)
Understory: measured: leaf and wood NPP of saplings (allometry for 1999-2000) and leaf and wood apical NPP of herbs/shrubs (harvest for 1999) – not measured: nonvascular NPP and NPP due to stem secondary growth of shrubs (both thought to be negligible; mosses covered only 1% ground)

Respiration[34]

Methods
Soil: chambers + infrared gas analyzer (4 to 5 times per year in 1999-2000); replicates: n=16
Soil – heterotrophic component: 53% of Rsoil from literature + Monte Carlo approach
Coarse woody debris: based on surveys and decomposition rate from literature

Scaling
Soil:
TEMPORAL: function of temperature and moisture
SPATIAL: basic calculation

SMEARII

Net primary production
Not available

Respiration[41]

Methods
Soil: automated chambers (hourly measurements, 2003-2006; n=3) + manual chambers (5-8 sampling periods per summer; n=14-20)
Stem: automated chambers (2003-2006, only on one trees, with one chamber below the crown and one in the crown; in summer 2003 the system was circulating among different trees and stem heights to capture variability)
Leaves/branches: automated chambers (whole years for 2003-2006); n=3-4 shoots
Understory: considered with soil
Coarse woody debris: not measured.

Scaling
Soil:
TEMPORAL: gaps in automated data filled with temperature functions (varying with seasonality)
SPATIAL: basic calculation
Stem:
TEMPORAL: gaps in automated data filled with temperature functions (varying with seasonality); lag between stem temperature and CO_2 efflux considered
SPATIAL: wood surface area (including BRANCHES)
Leaves:
TEMPORAL: gaps in automated data filled with temperature functions (varying with seasonality)
SPATIAL: leaf area needles

Sylvania hardwood

Net primary production [19,43]
Stem and branches: allometry, 2002-2003
Coarse roots: allometry, 2002-2003
Leaves: allometry + litter traps, 2002-2003
Fine roots: in-growth cores, 2002-2003
Understory: negligible

Respiration[19,44]

Methods
Soil: soil chambers + LI-6400 portable system (3–4 weeks in the 2002 and 2003 growing seasons); replicates: n=20 plots
Fine root respiration: Rs-Rh
Soil – heterotrophic component: no methodological info available (assumed lowest quality category)
Stem/branches: chambers + LI-6400 portable system (monthly in growing season 2002 and 2003); replicates: n=12-19 trees per 3 species. Only stem measured but assumed that branches had the same respiration rate as stem.
Leaves: detached leaves analyzed with LI-6400 portable system (June, July and August 2002 and 2003); replicates: n=20-30 leaves from 7-10 trees per 3 species.
Understory: negligible
Coarse woody debris: chambers + LI-6400 portable system (as for soil) on large debris (every 3–4 weeks during the growing season 2002 and 2003).

Scaling
Soil:
TEMPORAL: classical relationship with temperature and Q_{10} (seasonal and site data)
SPATIAL: basic calculation
Stem:
TEMPORAL: classical relationship with temperature and Q_{10} (seasonal and site data)
SPATIAL: sapwood volume
Leaves:
TEMPORAL: classical relationship with temperature and Q_{10} (seasonal and site data)
SPATIAL: dry biomass
Coarse woody debris:
TEMPORAL: classical relationship with temperature and Q_{10} (seasonal and site data)
SPATIAL: surface area

Takayama

Net primary production [45]
Stem and branches: allometry, 1999-2003
Coarse roots: allometry, 1999-2003
Leaves: litter traps, 1999-2003
Fine roots: minirhizotron + seasonal core sampling 2000
Understory: harvest of dominant understory species in 1993-1994 but trees with DBH<5 cm not measured

Respiration[45]

Methods
Soil: chambers + IRGA (measured continuously for 24-48 hours once or twice a month, 1999-2003); replicates: n=4
Soil – heterotrophic component: trenching experiment in 1999
Coarse woody debris: pool of coarse woody debris on the forest floor assumed in steady state; so decomposition= production (from surveys/litter traps)

Scaling
Soil:
TEMPORAL: temperature function
SPATIAL: taking into account comparison of the 4 chambers studied with 100 chambers in another study focused on soil heterogeneity at the site (note that site has ridges, valleys etc. so with topographical complexity)

Tapajos km 67

Net primary production (from[5] and references therein)
Stem and branches: allometry
Coarse roots: assumed some production-to-biomass ratio of aboveground wood and coarse root biomass being 21% of aboveground wood biomass
Leaves: litter traps
Fine roots: sequential coring (every two months for two years; 0-10 cm depth with correction for soil depth of 1.0 m using root biomass profiles, compartment flow model of Sanantonio and Grace 1987[105]); replicates: 6 plots for two soil types.
Understory: ground vegetation and trees <10 cm diameter not measured
Reproductive organs: litter traps
Branch turnover: surveys
VOC: empirical for similar sites
DOC: negligible

Respiration (from Malhi et al 2009[5] and cited references)

Methods
Soil: chambers: Keller et al 2005[106] (1.5 y with sampling at monthly interval, 08.00-18.00, n=8), Silver et al 2005[107] (07/1999-05/2001, 1-2.5 months interval, 6 plots on two soil types), Davidson et al in[5] (2000-2005, 5 times per year; n=18), Varner et al in[75]: no info.
Fine roots: steady-state mass balance approach based on quantifying above-ground and below-ground litter input, assuming that heterotrophic respiration rates are equal to litter input rates, and allocating the remaining soil respiration to root respiration; at the clay sites, the mass balance approach provided root respiration consistent with trenching approach[5].
Soil – heterotrophic component: Rsoil - Rroot
Stem: chambers + infrared gas analyzer (February, April, July, and October of 2004); replicates: 21 individual trees/large vines[108]
Leaves: Leaf dark respiration rates assessed from light-response curves from 68 leaves from 26 individuals (with photosynthetic gas exchange system LI-6400, morning hours 08.00-13.00)[109]. Photoinhibition equations of Atkin et al. (2000)[110] applied to these values and integrated throughout the canopy[5,111]. Total leaf respiration is the sum of nighttime leaf respiration and daytime leaf respiration.
Understory: ground vegetation and trees <10 cm diameter not measured
Coarse woody debris: derived from decay rate equation based on site measurements[112]

Scaling
Soil:
TEMPORAL: no info (likely average through year, no seasonality)
SPATIAL: taking into account two soil type and their proportion (32% and 68%)
Stem:
TEMPORAL: no info (likely average through year, no seasonality)
SPATIAL: wood surface area (considering also BRANCHES)
Leaves:
TEMPORAL: no info
SPATIAL: leaf area index

Thompson NSA NOBS

Net primary production
Stem and branches: allometry, 1999-2001[49]
Coarse roots: allometry, 1999-2001[49]
Leaves: allometry + litter traps, 1999-2001[49]
Fine roots: maximum-minimum soil core method for 2001 (difference between midsummer and autumnal biomass)[49]
Understory: apical growth harvested in 1999-2001[49], nonvascular growth measured in 1994[37] – secondary growth shrubs not measured[49]

Respiration

Methods
Soil: chambers + infrared gas analyzer on soil with mosses (monthly during the growing season between May 2001 and August 2002); replicates: n=8-16[48]
Soil – heterotrophic component: trenching experiment in 2001-2002[48]
Coarse woody debris: surveys and measured decomposition rates in 2000[47]

Scaling
Soil:
TEMPORAL: temperature function
SPATIAL: basic calculation

Tumbarumba

Net primary production [51]
Stem and branches: allometry, 2002-2003
Coarse roots: allometry, 2002-2003
Leaves: litter traps, 2002-2003
Fine roots: sequential coring, 2003
Understory: approximated as percentage of overstory NPP using data from a similar site

Respiration[51]

Methods (data from Nov 2001- Aug 2002 and March 2005)
Soil: chambers using absorption of CO_2 by soda lime (24-h period each month during the year); replicates: n=30 plots with 90 measurements per plot.
Fine root respiration: trenching (monthly measurements over one year) + chambers of intact fine roots in situ
Soil – heterotrophic component: Rsoil - Rroot
Stem: chambers with LI-6200 gas analysis system on different tree sizes
Branches: chambers with LI-6200 gas analysis system on different branch diameter classes
Coarse roots: chambers with LI-6200 gas analysis system on different root diameter classes
Leaves: leaf gas exchange system at night and day (with leaves covered by cloth) on saplings and mature trees (n=8) and on range of leaf ages and positions in the canopy
Understory: as for leaves
Coarse woody debris: not mentioned / not measured

Scaling
Soil:
TEMPORAL: Model of soil temperature, volumetric soil moisture content, and plot data for forest floor litter mass, rate of litterfall, total biomass, soil carbon content, plot number and month.
SPATIAL: Soil respiration calculated for each of four vegetation classes and total site respiration determined from the fraction of the total area occupied by each vegetation class.
Stem/branches/coarse roots:
TEMPORAL temperature response function
SPATIAL: sapwood volume
Leaves:
TEMPORAL: temperature response function
SPATIAL: leaf area index
Understory:
TEMPORAL: temperature response function
SPATIAL: leaf area index
Roots:
TEMPORAL: temperature response function (seasonal and site data)
SPATIAL: Respiration rate per root surface area multiplied by specific root area and fine root mass per hectare, and seasonal variation in root mass

TurkeyPointTP02

Net primary production [54]
Stem and branches: allometry + accounting branch/tree mortality, 2006
Coarse roots: allometry, 2006
Leaves: allometry+ litter traps, 2006
Fine roots: fine root biomass stock + fine root turnover rate as average of three methods: mass balance approach of Raich and Nadelhoffer (1989)[103], relationship between available N from mineralization following Aber et al. (1985)[104], and fixed turnover rate (0.60 yr^{-1}) from the literature.
Understory: allometry + harvesting for grasses, 2006
Herbivory: from literature, 2006

Respiration[53 54]

Methods
Soil: chambers + LI-6400 photosynthesis system (monthly basis from 1 January 2006 to 31 December 2006); replicates: n=12[53]
Fine root respiration: derived from ratio Rroot: Rh-soil from trenching experiment[54] and total soil respiration[53]
Soil – heterotrophic component: Rsoil - Rroot
Stem: assumed same respiration rate as in TurkeyPointTP89 (see below)
Leaves: dark foliar gas exchange measurement (i.e. net CO_2 exchange of foliage at zero light level) with small chamber (for 10-15 needles 1-year-old) and LI-6400 to generate the light response curves (monthly basis from June to August in 2006 and additional in April, May, September, and November in 2007); in mid-canopy, replicates not reported. Interannual variability of leaf respiration was assumed to be small[53].
Understory: not measured
Coarse woody debris: debris stock mass multiplied by a decomposition rate from literature[54]

Scaling
Soil[53]:
TEMPORAL: model between respiration and temperature, precipitation, mean thickness of the forest floor horizon (cm) and carbon-to-nitrogen ratio of the forest floor
SPATIAL: basic calculation
Stem[53]:
TEMPORAL :model between respiration and temperature, precipitation and DBH
SPATIAL: sapwood volume (considering also BRANCHES)
Leaves[53]:
TEMPORAL: model between respiration and temperature, precipitation, VPD and PAR
SPATIAL: surface area of needles / LAI
Soil – heterotrophic component[54]:
TEMPORAL: model between respiration and temperature
SPATIAL: basic calculation

TurkeyPointTP89

Net primary production [54]
Stem and branches: allometry + accounting branch/tree mortality, 2006
Coarse roots: allometry, 2006
Leaves: allometry+ litter traps, 2006
Fine roots: fine root biomass stock + fine root turnover rate as average of three methods: mass balance approach of Raich and Nadelhoffer (1989)[103], relationship between available N from mineralization following Aber et al. (1985)[104], and fixed turnover rate (0.60 yr^{-1}) from the literature.
Understory: allometry + harvesting for grasses, 2006
Herbivory: from literature, 2006

Respiration [53,54]

Methods
Soil: chambers + LI-6400 photosynthesis system (monthly basis from 1 January 2006 to 31 December 2006); replicates: n=12[53]
Fine root respiration: derived from ratio Rroot: Rh-soil from trenching experiment[54] and total soil respiration[53]
Soil – heterotrophic component: Rsoil - Rroot
Stem: soil chambers + LI-6400 (monthly basis, from April to November 2006) [53]
Leaves: dark foliar gas exchange measurement (i.e. net CO_2 exchange of foliage at zero light level) with small chamber (for 10-15 needles 1-year-old) and LI-6400 to generate the light response curves (monthly basis from June to August in 2006); mid-canopy for 2-3 trees. Interannual variability of leaf respiration was assumed to be small[53]
Understory: negligible
Coarse woody debris: debris stock mass multiplied by a decomposition rate from literature[54]

Scaling
Soil[53]:
TEMPORAL: model between respiration and temperature, precipitation, mean thickness of the forest floor horizon (cm) and carbon - to - nitrogen ratio of the forest floor
SPATIAL: basic calculation
Stem [53]:
TEMPORAL :model between respiration and temperature, precipitation and DBH
SPATIAL: sapwood volume (considering also BRANCHES)
Leaves [53]:
TEMPORAL: model between respiration and temperature, precipitation, VPD and PAR
SPATIAL: surface area of needles / LAI
Soil – heterotrophic component[54]:
TEMPORAL: model between respiration and temperature
SPATIAL: basic calculation

TurkeyPointTP74

Net primary production [54]
Stem and branches: allometry + accounting branch/tree mortality, 2006
Coarse roots: allometry, 2006
Leaves: allometry+ litter traps, 2006
Fine roots: fine root biomass stock + fine root turnover rate as average of three methods: mass balance approach of Raich and Nadelhoffer (1989)[103], relationship between available N from mineralization following Aber et al. (1985)[104], and fixed turnover rate (0.60 yr^{-1}) from the literature.
Understory: allometry + harvesting for grasses, 2006
Herbivory: from literature, 2006

Respiration[53,54]

Methods
Soil: chambers + LI-6400 photosynthesis system (monthly basis from 1 January 2006 to 31 December 2006); replicates: n=12[53]
Fine root respiration: derived from ratio Rroot: Rh soil from trenching experiment[54] and total soil respiration[53]
Soil – heterotrophic component: Rsoil - Rroot
Stem: soil chambers + LI-6400 (monthly basis, from April to November 2006)[53]
Leaves: dark foliar gas exchange measurement (i.e. net CO_2 exchange of foliage at zero light level) with small chamber (for 10-15 needles 1-year-old) and LI-6400 to generate the light response curves (monthly basis from June to August in 2006); mid-canopy for 2-3 trees. Interannual variability of leaf respiration was assumed to be small[53]
Understory: not measured
Coarse woody debris: debris stock mass multiplied by a decomposition rate from literature[54]

Scaling
Soil [53]:
TEMPORAL: model between respiration and temperature, precipitation, mean thickness of the forest floor horizon (cm) and carbon-to-nitrogen ratio of the forest floor
SPATIAL: basic calculation
Stem [53]:
TEMPORAL :model between respiration and temperature, precipitation and DBH
SPATIAL: sapwood volume (considering also BRANCHES)
Leaves [53]:
TEMPORAL: model between respiration and temperature, precipitation, VPD and PAR
SPATIAL: surface area of needles / LAI
Soil – heterotrophic component [54]:
TEMPORAL: model between respiration and temperature
SPATIAL: basic calculation

TurkeyPointTP39

Net primary production [54]
Stem and branches: allometry + accounting branch/tree mortality, 2006
Coarse roots: allometry, 2006
Leaves: allometry+ litter traps, 2006
Fine roots: fine root biomass stock + fine root turnover rate as average of three methods: mass balance approach of Raich and Nadelhoffer (1989)[103], relationship between available N from mineralization following Aber et al. (1985)[104], and fixed turnover rate (0.60 yr^{-1}) from the literature.
Understory: allometry + harvesting for grasses, 2006
Herbivory: from literature, 2006

Respiration[53 54]

Methods
Soil: chambers + LI-6400 photosynthesis system (monthly basis from 1 January 2006 to 31 December 2006); replicates: n=12[53]
Fine root respiration: derived from ratio Rroot: Rh-soil from trenching experiment[54] and total soil respiration[53]
Soil – heterotrophic component: Rsoil - Rroot
Stem: soil chambers + LI-6400 (monthly basis, from April to November 2006)[53]
Leaves: dark foliar gas exchange measurement (i.e. net CO_2 exchange of foliage at zero light level) with small chamber (for 10-15 needles 1-year-old) and LI-6400 to generate the light response curves (monthly basis from June to August in 2006; additionally in April, May, September, and November in 2007); mid-canopy for 2-3 trees. Interannual variability of leaf respiration was assumed to be small[53]
Understory: not measured
Coarse woody debris: debris stock mass multiplied by a decomposition rate from literature[54]

Scaling
Soil [53]:
TEMPORAL: model between respiration and temperature, precipitation, mean thickness of the forest floor horizon (cm) and carbon-to-nitrogen ratio of the forest floor
SPATIAL: basic calculation
Stem [53]:
TEMPORAL :model between respiration and temperature, precipitation and DBH
SPATIAL: sapwood volume (considering also BRANCHES)
Leaves [53]
TEMPORAL: model between respiration and temperature, precipitation, VPD and PAR
SPATIAL: surface area of needles / LAI
Soil – heterotrophic component [54]:
TEMPORAL: model between respiration and temperature
SPATIAL: basic calculation

University of Michigan

Net primary production[55]
Stem and branches: allometry
Coarse roots: allometry
Leaves: litter traps
Fine roots: standing fine root biomass + turnover, with turnover as average of 3 methods: (1) from minirhizotron at the site, (2) N model of Aber et al. (1985)[104] and (3) mass balance approach of Raich and Nadelhoffer (1989)[103]
Understory: WOOD: allometry but saplings < 10 cm DBH not measured – LEAVES: allometry
Branch turnover: measured through surveys of net coarse woody debris production
Herbivory: considered

Respiration

Methods
Soil: chambers + gas analyzer (LI-COR LI-6400) (along year and also with snow with frequency every 3 days in summer to every 30 days in winter); replicates: n=3 collars in 8 plots[56]
Fine roots: Rs – Rh (see below)
Stem: chambers + gas analyzer (multiple times along 1999-2001); replicates: n=1-9 trees of 5 species[56]
Leaves: MAINTENACE: gas exchange detached leaves at night on fully expanded leaves (in 1999-2001 on multiple times varying from 1 to 9 per species); replicates: 6 leaves in upper and 6 leaves in lower canopy of 2-3 trees for 4 overstory species and 2 understory species. GROWTH: from biomass and fixed coefficient[56]
Understory: WOOD: not measured; LEAVES: measured[56]
Soil – heterotrophic component: using the component integration method, with soil taken from the site and analyzed for respiration in laboratory[55]
Coarse woody debris: not measured.

Scaling[56]
Soil:
TEMPORAL: function of temperature and soil water content developed for three periods (early season, late season, winter)
SPATIAL: test between measuring plots and entire EC footprint
Stem:
TEMPORAL: function of temperature and developed for three periods (early season, late season, winter) with uncommon formulation for equations
SPATIAL: sapwood volume (including BRANCHES)
Leaves:
TEMPORAL: function of temperature
SPATIAL: leaf area

Walker Range

Net primary production[57,58]
Stem and branches: allometry
Coarse roots: assumed 20% of aboveground wood increment
Leaves: litter traps
Fine roots: minirhizotron + fine root biomass
Understory: saplings: allometry; herbs: negligible
Reproductive material: accounted for
Herbivory: accounted for
Twigs: accounted for
VOC: negligible

Respiration (from Edwards and Hanson 2003[58] *and Hanson et al 2003*[57]*; otherwise indicated)*

Methods
Soil: chamber + gas analyzer (weekly or be-weekly 1992-1999); replicates: n=30
Fine roots: as 50% of soil respiration from generic/general relationships[97]
Soil – heterotrophic component: Rsoil - Rroots
Stem: chambers + infrared gas analysis. OVERSTORY: measurements taken through the year for both growing and non-growing season from 8.00-16.00 h; replicates: 6 trees for 3 species in 1993-1996, 6 trees for 1 species 1998-2000, with trees with different DBH on both north/south side. UNDERSTORY: measurements through the year; replicates: 10 trees for 2 species in 1994-1995. Estimates MAINTENANCE and GROWTH respiration both available based on non-growing season data (when only maintenance) applied to growing season (when both maintenance and growth) and/or growth derived from tissue construction factor.
Branches: chambers + infrared gas analysis (both for growing and non-growing season in 1997-1999); branches 1 year-old with diameter 1-2 cm; 1-2 branches of 1-3 trees of 3 species. Estimates MAINTENANCE and GROWTH respiration both available as for stem (see above).
Leaves: MAINTENANCE: chambers + infrared analysis on cut branches and/or in situ with dark chambers (measurements late summer after completion leaf growth in 1995, 1997 and 1999; n=3-40 leaves per species from mid-canopy, 4 overstory and 3 understory species); GROWTH: dry mass multiplied by factor
Understory: accounted for (see above in stem/leaves)
Corse woody debris: assumed 10% of dead wood pool

Scaling (from Edwards and Hanson 2003[58] *and Hanson et al 2003*[57]*, otherwise indicated)*
Soil:
TEMPORAL: based on temperature and soil water[113]
SPATIAL: basic calculation
Stem/branches:
TEMPORAL: temperature response function based on Q_{10} at the site
SPATIAL: wood volume
Leaves
TEMPORAL: temperature response function based on Q_{10} at a nearby similar site
SPATIAL: LAI

Willow Creek

Net primary production
Stem and branches: allometry, 1989-1999[20]
Coarse roots: 20% above ground wood production[20]
Leaves: litter traps, 1989-1999[20]
Fine roots: average of two methods: empirical model of Aber et al. 1985[20,104] and measured standing stock + turnover from similar sites[59]
Understory: allometry for woody plants + harvest for herbaceous plants[20]

Respiration

Methods
Soil: chambers + infrared gas analyzer (IRGA, LiCor) (monthly when the ground was not snow-covered in 2001-2002); replicates: n=32[59]
Fine roots: empirical model based on temperature and root tissue N concentration for similar sites[59,114]
Soil – heterotrophic component: Rsoil - Rroots
Stem: chambers + infrared gas analyzer (from May to November 2002); replicates: 20 trees (6-8 for 3 species) of various DBH, random azimuth[59]
Coarse roots: not measured
Leaves: MAINTENANCE: gas exchange system on cut branches performed in dark (period full leaf expansion – thus mid-late summer, measured at predawn); replicates: 40 leaves per species for 3 dominant species, leaves from low, mid and high positions in the canopy; GROWTH: mass-based empirical model[59]
Understory: not measured
Coarse woody debris: chambers and ground survey[60]

Scaling
Soil:
TEMPORAL: model dependent on temperature and soil water content based on site measurements[59]
SPATIAL: basic calculation
Stem:
TEMPORAL: classical Q_{10} model dependent on temperature and based on site measurements[59]
SPATIAL: sapwood volume (considering also BRANCHES)
Leaves:
TEMPORAL: Q_{10} model dependent on temperature and also with acclimation[59,91]
SPATIAL: leaf biomass

Wind River

Net primary production[62]
Stem and branches: allometry, 1995-1999
Coarse roots: allometry, 1995-1999
Leaves: litter traps, 1997-1999
Fine roots: standing biomass + turnover measured on site (data period 1999-2000)
Understory: accounted for
Herbivory: accounted for in 1995, 1996 and 1999
Epiphytes: accounted for

Respiration[62]

Methods
Soil: chambers + infrared gas analyzer (April, June, August and October 1997 and January 1998); replicates: n=8, probably done for portions with bare soil and no living roots.
Soil – heterotrophic component: component integration method[97]. Rh-soil divided into three portions: (i) litter (measured: standing biomass and decomposition rate with litterbags or literature), (ii) dead roots (measured: standing biomass and decomposition rate with litterbags or literature) and (iii) mineral soil (respiration mineral soil = Rsoil – respiration litter - respiration dead roots)
Coarse woody debris: surveys + decomposition derived in situ from difference in standing dead wood at two different times; respiration coarse woody debris includes also respiration of stem hart-rot obtained from surveys and assuming same decomposition rate for coarse woody debris.

Scaling:
Soil:
TEMPORAL: not accounted for
SPATIAL: amount of soil

Wytham Woods

Net primary production[63]
Stem and branches: allometry, 2008
Coarse roots: assumed 20% of aboveground woody production
Leaves: litter traps, 2008
Fine roots: indirectly derived for 2008 from total belowground C allocation method (TBCA) having/assuming all other components
Understory: not measured
Reproductive material: litter traps, 2008

Respiration[63]

Methods
Soil: soil chambers + portable infra-red gas analysis (IRGA) (monthly per year); replicates: n=30-35.
Fine roots: Rsoil – Rh-soil
Soil – heterotrophic component: root exclusion method: measurements on bags with mesh size stopping roots (April-Nov 2008)[115]
Stem: adapted soil chambers + portable infra-red gas analysis (IRGA) (monthly from April to November 2008 always same moment, the morning); replicates: n=8 for 2 species.
Leaves: no info on method (measurements on one occasion in 2001, night); replicates: 20 measurements (for shade and sun leaves) on 5 trees for 2 dominant species (data for other species derived from data for dominant species); PHOTOINHIBITION accounted for
Understory: not measured
Rh CWD: MAG – FCWD, with MAG the mean annual production of aboveground coarse woody debris (CWD) and FCWD is the CWD fraction entering the soil

Scaling
Soil:
TEMPORAL: fixed: monthly values determined from measurements during that month
SPATIAL: basic calculation
Stem:
TEMPORAL: fixed: monthly values determined from measurements during that month, with November taken for the winter period
SPATIAL: stem area (assumed based on literature) with branches accounted for
Leaves:
TEMPORAL: relationship with temperature with Q_{10} assumed as equal to 2
SPATIAL: leaf area index

Xishuangbanna

Net primary production[65]
Stem and branches: allometry, 2003-2006
Coarse roots: allometry, 2003-2006
Leaves: litter traps, 2003-2006
Fine roots: sequential soil coring (no info about years)
Understory: allometry+ litter traps, 2003-2006
Reproductive material: litter traps, 2003-2006
Twigs: litter traps, 2003-2006
Epiphytes: litter traps, 2003-2006

Respiration[65,116]

Methods
Soil: chambers + gas chromatographic analysis (once a week at 09.00-11.00 am for whole year, 2003-2007); replicates: n=6.
Fine root respiration: Rsoil – Rh-soil
Soil – heterotrophic component: trenching
Stem: chambers + infrared gas analyzer Li-820 LI-COR (January, April, June and October, no info on years); replicates: 5 trees for 10 species (north and south faces separately).
Leaves: portable photosynthesis system Li-6400 (January, April, June and October, no info on years); replicates: 3 trees, 3 layers per trees, and 3 leaves per layer + 3 leaves on saplings.
Coarse roots: respiration per unit root biomass assumed equal as the one of stem biomass
Understory: comprised in other compartments (Stem and Leaves)
Coarse woody debris: empirical relationship (Rh-cwd = k × (total cwd carbon density)) independent to decay class and other environmental factors and with parameters from literature

Scaling
Soil:
TEMPORAL: function temperature[116]
SPATIAL: basic calculation
Stem:
TEMPORAL: temperature response function
SPATIAL: sapwood volume
Leaves:
TEMPORAL: temperature response function
SPATIAL: LAI
Coarse woody debris:
TEMPORAL: fixed (see above)
SPATIAL: derived from inventory of standing dead wood

Yamashiro

Net primary production[66]
Stem and branches: allometry, 1999-2003
Coarse roots: allometry, 1999-2003
Leaves: litter traps, 1999-2003
Fine roots: indirectly derived from site measurements of soil C fluxes
Understory: allometry for woody vegetation and destructive sampling for herbaceous vegetation
Branch turnover: taken into account

Respiration[66]

Methods
Soil: chambers + IRGA (from one to four times per month, for a total of 74 times from July 2002 to May 2003; extra measurements on 4 occasions in summer 2002 on a larger sample size); replicates: n=96 (for entire period) and n=264 (for 4 occasions in summer 2002).
Fine roots: automated chambers + IRGA; measurements in situ on attached roots from April 2004 to September 2005
Soil – heterotrophic component: Rsoil - Rroot
Coarse woody debris: surveys + respiration measurements

Scaling
Soil:
TEMPORAL: function of temperature and water content
SPATIAL: basic calculation
Fine roots:
TEMPORAL: function temperature[117]
SPATIAL: relationship between Rroot and root diameter, using probably root biomass
Coarse woody debris:
TEMPORAL: function based on temperature and moisture
SPATIAL: debris size and C density

Yatir

Net primary production
No data available meeting quality standard for our analysis

Respiration

Methods (in our analysis data used from October 2001 to September 2002)
Soil: chambers + LI-6400 system (from October 2000 to September 2006; regular measurements performed between midday and early afternoon, while 16- to 24-h cycles measurements carried out periodically); replicates: n=29 collars[67]
Fine roots: not available
Soil – heterotrophic component: not available
Stem: chambers + LI-6400 analyzer (46 occasions between March 2002 and April 2005, in afternoon between 14:00 and 16:00; on some days repeated measurements made at different times through the diurnal cycle); replicates: n=12 trees[68]
Leaves: LI6400-05 Conifer Chamber + LI-COR LI6400 portable photosynthesis system (measurements between March 2002 and October 2004, mainly in night); replicates: n=6-12 trees, measurements on current and previous year needles[68]
Understory: not measured
Coarse woody debris: not measured

Scaling
Soil[67]:
TEMPORAL: relationship with water content, temperature and PPFD
SPATIAL: corrected for rock-covered surface area
Stem[68]:
TEMPORAL: temperature response function
SPATIAL: wood area (considering also BRANCHES)
Leaves[68]:
TEMPORAL: temperature response
SPATIAL: projected leaf area

Supplementary References

1 South, A. rworldmap: A New R package for Mapping Global Data. *The R Journal* **3/1**, 35-43 (2011).

2 The R Core Team. *R: A language and environment for statistical computing*. (R Foundation for Statistical Computing, 2016).

3 Carswell, F. E. *et al.* Seasonality in CO_2 and H_2O flux at an eastern Amazonian rain forest. *Journal of Geophysical Research-Atmospheres* **107**, doi:10.1029/2000jd00284 (2002).

4 Doughty, C. E. *et al.* The production, allocation and cycling of carbon in a forest on fertile terra preta soil in eastern Amazonia compared with a forest on adjacent infertile soil. *Plant Ecology & Diversity* **7**, 41-53, doi:10.1080/17550874.2013.798367 (2014).

5 Malhi, Y. *et al.* Comprehensive assessment of carbon productivity, allocation and storage in three Amazonian forests. *Global Change Biology* **15**, 1255-1274, doi:10.1111/j.1365-2486.2008.01780.x (2009).

6 Wang, M., Guan, D.-X., Han, S.-J. & Wu, J.-L. Comparison of eddy covariance and chamber-based methods for measuring CO_2 flux in a temperate mixed forest. *Tree Physiology* **30**, 149-163, doi:10.1093/treephys/tpp098 (2010).

7 FLUXNET. <http://fluxnet.fluxdata.org/data/fluxnet2015-dataset/> (2015).

8 Bergeron, O., Margolis, H. A., Coursolle, C. & Giasson, M.-A. How does forest harvest influence carbon dioxide fluxes of black spruce ecosystems in eastern North America? *Agricultural and Forest Meteorology* **148**, 537-548, doi:10.1016/j.agrformet.2007.10.012 (2008).

9 Hermle, S., Lavigne, M. B., Bernier, P. Y., Bergeron, O. & Pare, D. Component respiration, ecosystem respiration and net primary production of a mature black spruce forest in northern Quebec. *Tree Physiology* **30**, 527-540, doi:10.1093/treephys/tpq002 (2010).

10 EuropeanFluxesDatabaseCluster. <http://www.europe-fluxdata.eu/> (2015).

11 Guidolotti, G., Rey, A., D'Andrea, E., Matteucci, G. & De Angelis, P. Effect of environmental variables and stand structure on ecosystem respiration components in a Mediterranean beech forest. *Tree Physiology* **33**, 960-972, doi:10.1093/treephys/tpt065 (2013).

12 Black, K. *et al.* Inventory and eddy covariance-based estimates of annual carbon sequestration in a Sitka spruce (*Picea sitchensis* (Bong.) Carr.) forest ecosystem. *European Journal of Forest Research* **126**, 167-178, doi:10.1007/s10342-005-0092-4 (2007).

13 Hamilton, J. G. *et al.* Forest carbon balance under elevated CO_2. *Oecologia* **131**, 250-260, doi:10.1007/s00442-002-0884-x (2002).

14 Novick, K. A. *et al.* On the difference in the net ecosystem exchange of CO_2 between deciduous and evergreen forests in the southeastern United States. *Global Change Biology* **21**, 827-842, doi:10.1111/gcb.12723 (2015).

15 Pritchard, S. G. *et al.* Long-term dynamics of mycorrhizal root tips in a loblolly pine forest grown with free-air CO_2 enrichment and soil N fertilization for 6 years. *Global Change Biology* **20**, 1313-1326, doi:10.1111/gcb.12409 (2014).

16 Mizoguchi, Y. *et al.* Seasonal and interannual variation in net ecosystem production of an evergreen needleleaf forest in Japan. *Journal of Forest Research* **17**, 283-295, doi:10.1007/s10310-011-0307-0 (2012).

17 Ohtsuka, T., Negishi, M., Sugita, K., Iimura, Y. & Hirota, M. Carbon cycling and sequestration in a Japanese red pine (*Pinus densiflora*) forest on lava flow of Mt. Fuji. *Ecological Research* **28**, 855-867, doi:10.1007/s11284-013-1067-4 (2013).

18 Kutsch, W. L. *et al.* Heterotrophic soil respiration and soil carbon dynamics in the deciduous Hainich forest obtained by three approaches. *Biogeochemistry* **100**, 167-183, doi:10.1007/s10533-010-9414-9 (2010).
19 Luyssaert, S. *et al.* CO_2 balance of boreal, temperate, and tropical forests derived from a global database. *Global Change Biology* **13**, 2509-2537, doi:10.1111/j.1365-2486.2007.01439.x (2007).
20 Curtis, P. S. *et al.* Biometric and eddy-covariance based estimates of annual carbon storage in five eastern North American deciduous forests. *Agricultural and Forest Meteorology* **113**, 3-19, doi:10.1016/s0168-1923(02)00099-0 (2002).
21 Davi, H. *et al.* Modelling carbon and water cycles in a beech forest Part II: Validation of the main processes from organ to stand scale. *Ecological Modelling* **185**, 387-405, doi:10.1016/j.ecolmodel.2005.01.003 (2005).
22 Epron, D., Farque, L., Lucot, E. & Badot, P. M. Soil CO_2 efflux in a beech forest: the contribution of root respiration. *Annals of Forest Science* **56**, 289-295, doi:10.1051/forest:19990403 (1999).
23 Granier, A., Bréda, N., Longdoz, B., Gross, P. & Ngao, J. Ten years of fluxes and stand growth in a young beech forest at Hesse, North-eastern France. *Annals of Forest Science* **65**, doi:10.1051/forest:2008052 (2008).
24 Granier, A. *et al.* The carbon balance of a young beech forest. *Functional Ecology* **14**, 312-325, doi:10.1046/j.1365-2435.2000.00434.x (2000).
25 Zha, T., Xing, Z., Wang, K.-Y., Kellomaki, S. & Barr, A. G. Total and component carbon fluxes of a Scots pine ecosystem from chamber measurements and eddy covariance. *Annals of Botany* **99**, 345-353, doi:10.1093/aob/mcl266 (2007).
26 Etzold, S. *et al.* The carbon balance of two contrasting mountain forest ecosystems in Switzerland: similar annual trends, but seasonal differences. *Ecosystems* **14**, 1289-1309, doi:10.1007/s10021-011-9481-3 (2011).
27 Chambers, J. Q. *et al.* Respiration from a tropical forest ecosystem: Partitioning of sources and low carbon use efficiency. *Ecological Applications* **14**, S72-S88 (2004).
28 Thomas, C. K., Martin, J. G., Law, B. E. & Davis, K. Toward biologically meaningful net carbon exchange estimates for tall, dense canopies: Multi-level eddy covariance observations and canopy coupling regimes in a mature Douglas-fir forest in Oregon. *Agricultural and Forest Meteorology* **173**, 14-27, doi:10.1016/j.agrformet.2013.01.001 (2013).
29 Law, B. E. *et al.* Environmental controls over carbon dioxide and water vapor exchange of terrestrial vegetation. *Agricultural and Forest Meteorology* **113**, 97-120, doi:10.1016/s0168-1923(02)00104-1 (2002).
30 Law, B. E., Ryan, M. G. & Anthoni, P. M. Seasonal and annual respiration of a ponderosa pine ecosystem. *Global Change Biology* **5**, 169-182, doi:10.1046/j.1365-2486.1999.00214.x (1999).
31 Law, B. E., Waring, R. H., Anthoni, P. M. & Aber, J. D. Measurements of gross and net ecosystem productivity and water vapour exchange of a Pinus ponderosa ecosystem, and an evaluation of two generalized models. *Global Change Biology* **6**, 155-168, doi:10.1046/j.1365-2486.2000.00291.x (2000).
32 Ehman, J. L. *et al.* An initial intercomparison of micrometeorological and ecological inventory estimates of carbon exchange in a mid-latitude deciduous forest. *Global Change Biology* **8**, 575-589, doi:10.1046/j.1365-2486.2002.00492.x (2002).
33 Dore, S. *et al.* Carbon and water fluxes from ponderosa pine forests disturbed by wildfire and thinning. *Ecological Applications* **20**, 663-683, doi:10.1890/09-0934.1 (2010).

34 Howard, E. A., Gower, S. T., Foley, J. A. & Kucharik, C. J. Effects of logging on carbon dynamics of a jack pine forest in Saskatchewan, Canada. *Global Change Biology* **10**, 1267-1284, doi:10.1111/j.1365-2486.2004.00804.x (2004).
35 Kljun, N. *et al.* Response of net ecosystem productivity of three boreal forest stands to drought (vol 9, pg 1128, 2006). *Ecosystems* **10**, 1039-1055, doi:10.1007/s10021-007-9088-x (2007).
36 Barr, A. G. *et al.* Climatic controls on the carbon and water balances of a boreal aspen forest, 1994-2003. *Global Change Biology* **13**, 561-576, doi:10.1111/j.1365-2486.2006.01220.x (2007).
37 Gower, S. T. *et al.* Carbon distribution and aboveground net primary production in aspen, jack pine, and black spruce stands in Saskatchewan and Manitoba, Canada. *Journal of Geophysical Research-Atmospheres* **102**, 29029-29041, doi:10.1029/97jd02317 (1997).
38 Russell, C. A. & Voroney, R. P. Carbon dioxide efflux from the floor of a boreal aspen forest. I. Relationship to environmental variables and estimates of C respired. *Canadian Journal of Soil Science* **78**, 301-310 (1998).
39 Ryan, M. G., Lavigne, M. B. & Gower, S. T. Annual carbon cost of autotrophic respiration in boreal forest ecosystems in relation to species and climate. *Journal of Geophysical Research-Atmospheres* **102**, 28871-28883, doi:10.1029/97jd01236 (1997).
40 Steele, S. J., Gower, S. T., Vogel, J. G. & Norman, J. M. Root mass, net primary production and turnover in aspen, jack pine and black spruce forests in Saskatchewan and Manitoba, Canada. *Tree Physiology* **17**, 577-587 (1997).
41 Kolari, P. *et al.* CO_2 exchange and component CO_2 fluxes of a boreal Scots pine forest. *Boreal Environment Research* **14**, 761-783 (2009).
42 Desai, A. R., Bolstad, P. V., Cook, B. D., Davis, K. J. & Carey, E. V. Comparing net ecosystem exchange of carbon dioxide between an old-growth and mature forest in the upper Midwest, USA. *Agricultural and Forest Meteorology* **128**, 33-55, doi:10.1016/j.agrformet.2004.09.005 (2005).
43 Tang, J. & Bolstad, P. V. Carbon allocation in an old-growth forest in the Great Lakes region of the United States *Proceeding 7th International Carbon Dioxide Conference* (2005).
44 Tang, J. *et al.* Ecosystem respiration and its components in an old-growth forest in the Great Lakes region of the United States. *Agricultural and Forest Meteorology* **148**, 171-185, doi:10.1016/j.agrformet.2007.08.008 (2008).
45 Ohtsuka, T., Mo, W., Satomura, T., Inatomi, M. & Koizumi, H. Biometric based carbon flux measurements and net ecosystem production (NEP) in a temperate deciduous broad-leaved forest beneath a flux tower. *Ecosystems* **10**, 324-334, doi:10.1007/s10021-007-9017-z (2007).
46 Saigusa, N., Yamamoto, S., Murayama, S. & Kondo, H. Inter-annual variability of carbon budget components in an AsiaFlux forest site estimated by long-term flux measurements. *Agricultural and Forest Meteorology* **134**, 4-16, doi:10.1016/j.agrformet.2005.08.016 (2005).
47 Bond-Lamberty, B., Wang, C. & Gower, S. T. Annual carbon flux from woody debris for a boreal black spruce fire chronosequence. *Journal of Geophysical Research-Atmospheres* **108**, doi:10.1029/2001jd000839 (2002).
48 Bond-Lamberty, B., Wang, C. K. & Gower, S. T. Contribution of root respiration to soil surface CO_2 flux in a boreal black spruce chronosequence. *Tree Physiology* **24**, 1387-1395 (2004).

49 Bond-Lamberty, B., Wang, C. K. & Gower, S. T. Net primary production and net ecosystem production of a boreal black spruce wildfire chronosequence. *Global Change Biology* **10**, 473-487, doi:10.1111/j.1529-8817.2003.0742.x (2004).
50 Dunn, A. L., Barford, C. C., Wofsy, S. C., Goulden, M. L. & Daube, B. C. A long-term record of carbon exchange in a boreal black spruce forest: means, responses to interannual variability, and decadal trends. *Global Change Biology* **13**, 577-590, doi:10.1111/j.1365-2486.2006.01221.x (2007).
51 Keith, H. *et al.* Multiple measurements constrain estimates of net carbon exchange by a *Eucalyptus* forest. *Agricultural and Forest Meteorology* **149**, 535-558, doi:10.1016/j.agrformet.2008.10.002 (2009).
52 Keith, H., van Gorsel, E., Jacobsen, K. L. & Cleugh, H. A. Dynamics of carbon exchange in a *Eucalyptus* forest in response to interacting disturbance factors. *Agricultural and Forest Meteorology* **153**, 67-81, doi:10.1016/j.agrformet.2011.07.019 (2012).
53 Khomik, M. *et al.* Relative contributions of soil, foliar, and woody tissue respiration to total ecosystem respiration in four pine forests of different ages. *Journal of Geophysical Research-Biogeosciences* **115**, doi:10.1029/2009jg001089 (2010).
54 Peichl, M., Brodeur, J. J., Khomik, M. & Arain, M. A. Biometric and eddy-covariance based estimates of carbon fluxes in an age-sequence of temperate pine forests. *Agricultural and Forest Meteorology* **150**, 952-965, doi:10.1016/j.agrformet.2010.03.002 (2010).
55 Gough, C. M., Vogel, C. S., Schmid, H. P., Su, H. B. & Curtis, P. S. Multi-year convergence of biometric and meteorological estimates of forest carbon storage. *Agricultural and Forest Meteorology* **148**, 158-170, doi:10.1016/j.agrformet.2007.08.004 (2008).
56 Curtis, P. S. *et al.* Respiratory carbon losses and the carbon-use efficiency of a northern hardwood forest, 1999-2003. *New Phytologist* **167**, 437-455, doi:10.1111/j.1469-8137.2005.01438.x (2005).
57 Hanson, P. J., Edwards, N. T., Tschaplinski, T. J., Wullschleger, S. D. & Joslin, J. D. in *North American temperate deciduous forest responses to changing precipitation regimes* (eds Paul J. Hanson & Stan D. Wullschleger) 378-395 (Springer-Verlag New York, 2003).
58 Edwards, N. T. & Hanson, P. J. in *North American temperate deciduous forest responses to changing precipitation regimes* (eds Paul J. Hanson & Stan D. Wullschleger) 48-66 (Springer-Verlag New York, 2003).
59 Bolstad, P. V., Davis, K. J., Martin, J., Cook, B. D. & Wang, W. Component and whole-system respiration fluxes in northern deciduous forests. *Tree Physiology* **24**, 493-504 (2004).
60 Cook, B. D. *et al.* Carbon exchange and venting anomalies in an upland deciduous forest in northern Wisconsin, USA. *Agricultural and Forest Meteorology* **126**, 271-295, doi:10.1016/j.agrformet.2004.06.008 (2004).
61 Falk, M., Wharton, S., Schroeder, M., Ustin, S. & U, K. T. P. Flux partitioning in an old-growth forest: seasonal and interannual dynamics. *Tree Physiology* **28**, 509-520 (2008).
62 Harmon, M. E. *et al.* Production, respiration, and overall carbon balance in an old-growth *Pseudotsuga-tsuga* forest ecosystem. *Ecosystems* **7**, 498-512, doi:10.1007/s10021-004-0140-9 (2004).
63 Fenn, K., Malhi, Y., Morecroft, M., Lloyd, C. & Thomas, M. The carbon cycle of a maritime ancient temperate broadleaved woodland at seasonal and annual scales. *Ecosystems* **18**, 1-15, doi:10.1007/s10021-014-9793-1 (2015).

64 Thomas, M. V. *et al.* Carbon dioxide fluxes over an ancient broadleaved deciduous woodland in southern England. *Biogeosciences* **8**, 1595-1613, doi:10.5194/bg-8-1595-2011 (2011).
65 Tan, Z. *et al.* Carbon balance of a primary tropical seasonal rain forest. *Journal of Geophysical Research-Atmospheres* **115**, doi:10.1029/2009jd012913 (2010).
66 Kominami, Y. *et al.* Biometric and eddy-covariance-based estimates of carbon balance for a warm-temperate mixed forest in Japan. *Agricultural and Forest Meteorology* **148**, 723-737, doi:10.1016/j.agrformet.2008.01.017 (2008).
67 Gruenzweig, J. M. *et al.* Water limitation to soil CO_2 efflux in a pine forest at the semiarid "timberline". *Journal of Geophysical Research-Biogeosciences* **114**, doi:10.1029/2008jg000874 (2009).
68 Maseyk, K., Grunzweig, J. M., Rotenberg, E. & Yakir, D. Respiration acclimation contributes to high carbon-use efficiency in a seasonally dry pine forest. *Global Change Biology* **14**, 1553-1567, doi:10.1111/j.1365-2486.2008.01604.x (2008).
69 Aragao, L. E. O. C. *et al.* Above- and below-ground net primary productivity across ten Amazonian forests on contrasting soils. *Biogeosciences* **6**, 2759-2778 (2009).
70 Luo, Y. *et al.* Gross primary productivity in Duke Forest: Modeling synthesis of CO2 experiment and eddy-flux data. *Ecological Applications* **11**, 239-252 (2001).
71 Hirata, R. *et al.* Spatial distribution of carbon balance in forest ecosystems across East Asia. *Agricultural and Forest Meteorology* **148**, 761-775, doi:10.1016/j.agrformet.2007.11.016 (2008).
72 Wang, K. Y., Kellomaki, S., Zha, T. & Peltola, H. Seasonal variation in energy and water fluxes in a pine forest: an analysis based on eddy covariance and an integrated model. *Ecological Modelling* **179**, 259-279, doi:10.1016/j.ecolmodel.2003.12.049 (2004).
73 Waring, R. *et al.* Why is the productivity of Douglas-fir higher in New Zealand than in its native range in the Pacific Northwest, USA? *Forest Ecology and Management* **255**, 4040-4046, doi:10.1016/j.foreco.2008.03.049 (2008).
74 Barr, A. G. *et al.* Inter-annual variability in the leaf area index of a boreal aspen-hazelnut forest in relation to net ecosystem production. *Agricultural and Forest Meteorology* **126**, 237-255, doi:10.1016/j.agrformet.2004.06.011 (2004).
75 Hutyra, L. R. *et al.* Seasonal controls on the exchange of carbon and water in an Amazonian rain forest. *Journal of Geophysical Research-Biogeosciences* **112**, doi:10.1029/2006jg000365 (2007).
76 Bond-Lamberty, B., Wang, C., Gower, S. T. & Norman, J. Leaf area dynamics of a boreal black spruce fire chronosequence. *Tree Physiology* **22**, 993-1001 (2002).
77 Leuning, R., Zhang, Y. Q., Rajaud, A., Cleugh, H. & Tu, K. A simple surface conductance model to estimate regional evaporation using MODIS leaf area index and the Penman-Monteith equation. *Water Resources Research* **44**, doi:10.1029/2007wr006562 (2008).
78 Shaw, D. C. *et al.* Ecological setting of the wind river old-growth forest. *Ecosystems* **7**, 427-439, doi:10.1007/s10021-004-0135-6 (2004).
79 Zhang, Y. *et al.* Annual variation of carbon flux and impact factors in the tropical seasonal rain forest of Xishuangbanna, SW China. *Science in China Series D-Earth Sciences* **49**, 150-162, doi:10.1007/s11430-006-8150-4 (2006).
80 Miyama, T., Kominami, Y., Tamai, K., Nobuhiro, T. & Goto, Y. Automated foliage chamber method for long-term measurement Of CO2 flux in the uppermost canopy. *Tellus Series B-Chemical and Physical Meteorology* **55**, 322-330, doi:10.1034/j.1600-0889.2003.00010.x (2003).

81 Volcani, A., Karnieli, A. & Svoray, T. The use of remote sensing and GIS for spatio-temporal analysis of the physiological state of a semi-arid forest with respect to drought years. *Forest Ecology and Management* **215**, 239-250, doi:10.1016/j.foreco.2005.05.063 (2005).
82 Gielen, B. *et al.* Biometric and eddy covariance-based assessment of decadal carbon sequestration of a temperate Scots pine forest. *Agricultural and Forest Meteorology* **174**, 135-143, doi:10.1016/j.agrformet.2013.02.008 (2013).
83 Barford, C. C. *et al.* Factors controlling long- and short-term sequestration of atmospheric CO_2 in a mid-latitude forest. *Science* **294**, 1688-1691, doi:10.1126/science.1062962 (2001).
84 Coursolle, C., Giasson, M.-A., Margolis, H. A. & Bernier, P. Y. Moving towards carbon neutrality: CO_2 exchange of a black spruce forest ecosystem during the first 10 years of recovery after harvest. *Canadian Journal of Forest Research-Revue Canadienne De Recherche Forestiere* **42**, 1908-1918, doi:10.1139/x2012-133 (2012).
85 Pyle, E. H. *et al.* Dynamics of carbon, biomass, and structure in two Amazonian forests. *Journal of Geophysical Research-Biogeosciences* **113**, doi:10.1029/2007jg000592 (2008).
86 Vogt, K. A., Grier, C. C., Meier, C. E. & Edmonds, R. L. Mycorrhizal role in net primary production and nutrient cycling in *Abies amabilis* ecosystems in Western Washington *Ecology* **63**, 370-380, doi:10.2307/1938955 (1982).
87 Finlay, R. D. & Söderström, B. in *Mycorrhizal functioning* (ed M. F. Allen) 134-160 (Chapman and Hall, 1992).
88 Hobbie, E. A. Carbon allocation to ectomycorrhizal fungi correlates with belowground allocation in culture studies. *Ecology* **87**, 563-569, doi:10.1890/05-0755 (2006).
89 Allen, M. F. & Kitajima, K. Net primary production of ectomycorrhizas in a California forest. *Fungal Ecology* **10**, 81-90, doi:10.1016/j.funeco.2014.01.007 (2014).
90 Allen, M. F. *et al.* Responses to chronic N fertilization of ectomycorrhizal pinon but not arbuscular mycorrhizal juniper in a pinon-juniper woodland. *Journal of Arid Environments* **74**, 1170-1176, doi:10.1016/j.jaridenv.2010.05.001 (2010).
91 Tjoelker, M. G., Oleksyn, J. & Reich, P. B. Modelling respiration of vegetation: evidence for a general temperature-dependent Q_{10}. *Global Change Biology* **7**, 223-230, doi:10.1046/j.1365-2486.2001.00397.x (2001).
92 Andrews, J. A. & Schlesinger, W. H. Soil CO_2 dynamics, acidification, and chemical weathering in a temperate forest with experimental CO_2 enrichment. *Global Biogeochemical Cycles* **15**, 149-162, doi:10.1029/2000gb001278 (2001).
93 Hamilton, J. G., Thomas, R. B. & Delucia, E. H. Direct and indirect effects of elevated CO_2 on leaf respiration in a forest ecosystem. *Plant Cell and Environment* **24**, 975-982, doi:10.1046/j.0016-8025.2001.00730.x (2001).
94 Mund, M. *et al.* The influence of climate and fructification on the inter-annual variability of stem growth and net primary productivity in an old-growth, mixed beech forest. *Tree Physiology* **30**, 689-704, doi:10.1093/treephys/tpq027 (2010).
95 McClaugherty, C. A., Aber, J. D. & Melillo, J. M. The role of fine roots in the organic matter and nitrogen budgets of two forested ecosystems. *Ecology* **63**, 1481-1490, doi:10.2307/1938874 (1982).
96 Davidson, E. A., Belk, E. & Boone, R. D. Soil water content and temperature as independent or confounded factors controlling soil respiration in a temperate mixed hardwood forest. *Global Change Biology* **4**, 217-227, doi:10.1046/j.1365-2486.1998.00128.x (1998).

97 Hanson, P. J., Edwards, N. T., Garten, C. T. & Andrews, J. A. Separating root and soil microbial contributions to soil respiration: A review of methods and observations. *Biogeochemistry* **48**, 115-146, doi:10.1023/a:1006244819642 (2000).
98 Epron, D., Farque, L., Lucot, E. & Badot, P. M. Soil CO_2 efflux in a beech forest: dependence on soil temperature and soil water content. *Annals of Forest Science* **56**, 221-226, doi:10.1051/forest:19990304 (1999).
99 Epron, D., Le Dantec, V., Dufrene, E. & Granier, A. Seasonal dynamics of soil carbon dioxide efflux and simulated rhizosphere respiration in a beech forest. *Tree Physiology* **21**, 145-152 (2001).
100 Damesin, C., Ceschia, E., Le Goff, N., Ottorini, J. M. & Dufrene, E. Stem and branch respiration of beech: from tree measurements to estimations at the stand level. *New Phytologist* **153**, 159-172, doi:10.1046/j.0028-646X.2001.00296.x (2002).
101 Chambers, J. Q., Schimel, J. P. & Nobre, A. D. Respiration from coarse wood litter in central Amazon forests. *Biogeochemistry* **52**, 115-131, doi:10.1023/a:1006473530673 (2001).
102 Ruehr, N. K. & Buchmann, N. Soil respiration fluxes in a temperate mixed forest: seasonality and temperature sensitivities differ among microbial and root-rhizosphere respiration. *Tree Physiology* **30**, 165-176, doi:10.1093/treephys/tpp106 (2010).
103 Raich, J. W. & Nadelhoffer, K. J. Belowground carbon allocation in forest ecosystems – global trends. *Ecology* **70**, 1346-1354, doi:10.2307/1938194 (1989).
104 Aber, J. D., Melillo, J. M., Nadelhoffer, K. J., McClaugherty, C. A. & Pastor, J. Fine root turnover in forest ecosystems in relation to quantity and form of nitrogen availability - a comparison of two methods *Oecologia* **66**, 317-321, doi:10.1007/bf00378292 (1985).
105 Santantonio, D. & Grace, J. C. Estimating fine-root production and turnover from biomass and decomposition data: a compartment–flow model. *Canadian Journal of Forest Research-Revue Canadienne De Recherche Forestiere* **17**, 900-908, doi:10.1139/x87-141 (1987).
106 Keller, M. *et al.* Soil-atmosphere exchange of nitrous oxide, nitric oxide, methane, and carbon dioxide in logged and undisturbed forest in the Tapajos National Forest, Brazil. *Earth Interactions* **9** (2005).
107 Silver, W. L. *et al.* Fine root dynamics and trace gas fluxes in two lowland tropical forest soils. *Global Change Biology* **11**, 290-306, doi:10.1111/j.1365-2486.2005.00903.x (2005).
108 Nepstad, D. C. *et al.* The effects of partial throughfall exclusion on canopy processes, aboveground production, and biogeochemistry of an Amazon forest. *Journal of Geophysical Research-Atmospheres* **107**, doi:10.1029/2001jd000360 (2002).
109 Domingues, T. F., Berry, J. A., Martinelli, L. A., Ometto, J. P. H. B. & Ehleringer, J. R. Parameterization of canopy structure and leaf-level gas exchange for an eastern Amazonian tropical rain forest (Tapajos National Forest, Para, Brazil). *Earth Interactions* **9** (2005).
110 Atkin, O. K., Evans, J. R., Ball, M. C., Lambers, H. & Pons, T. L. Leaf respiration of snow gum in the light and dark. interactions between temperature and irradiance. *Plant Physiology* **122**, 915-923, doi:10.1104/pp.122.3.915 (2000).
111 Lloyd, J. *et al.* Optimisation of photosynthetic carbon gain and within-canopy gradients of associated foliar traits for Amazon forest trees. *Biogeosciences* **7**, 1833-1859, doi:10.5194/bg-7-1833-2010 (2010).
112 Hutyra, L. R. *et al.* Resolving systematic errors in estimates of net ecosystem exchange of CO_2 and ecosystem respiration in a tropical forest biome. *Agricultural and Forest Meteorology* **148**, 1266-1279, doi:10.1016/j.agrformet.2008.03.007 (2008).

113 Hanson, P. J. *et al.* in *North American temperate deciduous forest responses to changing precipitation regimes* (eds Paul J. Hanson & Stan D. Wullschleger) 163-189 (Springer-Verlag New York, 2003).
114 Zogg, G. P., Zak, D. R., Burton, A. J. & Pregitzer, K. S. Fine root respiration in northern hardwood forests in relation to temperature and nitrogen availability. *Tree Physiology* **16**, 719-725 (1996).
115 Fenn, K. M., Malhi, Y. & Morecroft, M. D. Soil CO_2 efflux in a temperate deciduous forest: Environmental drivers and component contributions. *Soil Biology & Biochemistry* **42**, 1685-1693, doi:10.1016/j.soilbio.2010.05.028 (2010).
116 Sha, L. Q. *et al.* Soil respiration in tropical seasonal rain forest in Xishuangbanna, SW China. *Science in China Series D-Earth Sciences* **48**, 189-197, doi:10.1360/05zd0019 (2005).
117 Dannoura, M. *et al.* Development of an automatic chamber system for long-term measurements of CO_2 flux from roots. *Tellus Series B-Chemical and Physical Meteorology* **58**, 502-512, doi:10.1111/j.1600-0889.2006.00216.x (2006).
